# NOTUM promotes thermogenic capacity and protects against diet-induced obesity in male mice

**DOI:** 10.1038/s41598-021-95720-1

**Published:** 2021-08-12

**Authors:** Fangfei Guo, Marcus Seldin, Miklós Péterfy, Sarada Charugundla, Zhiqiang Zhou, Stephen D. Lee, Alice Mouton, Prashant Rajbhandari, Wenchao Zhang, Matteo Pellegrini, Peter Tontonoz, Aldons J. Lusis, Diana M. Shih

**Affiliations:** 1 Department of Microbiology, Immunology, and Molecular Genetics, Division of Cardiology, Department of Medicine, Department of Human Genetics, University of California, 10833 Le Conte Avenue, A2-237 CHS, Los Angeles, CA 90095-1679 USA; 2 Department of Biological Chemistry and Center for Epigenetics and Metabolism, University of California, Irvine, CA 92697 USA; 3 Department of Basic Medical Sciences, Western University of Health Sciences, Pomona, CA 91766 USA; 4 Department of Pathology and Laboratory Medicine, University of California, Los Angeles, CA 90095 USA; 5 Department of Ecology and Evolutionary Biology, University of California, Los Angeles, CA 90095 USA; 6 Diabetes, Obesity, and Metabolism Institute, Icahn School of Medicine Mount Sinai, New York, NY 10029 USA; 7 The Key Laboratory of Cardiovascular Remodeling and Function Research, Chinese Ministry of Education, Chinese National Health Commission and Chinese Academy of Medical Sciences, The State and Shandong Province Joint Key Laboratory of Translational Cardiovascular Medicine, Department of Cardiology, Qilu Hospital, Cheeloo College of Medicine, Shandong University, Jinan, 250012 Shandong China; 8 Department of Critical Care Medicine, Qilu Hospital, Cheeloo College of Medicine, Shandong University, Jinan, 250012 Shandong China; 9 Molecular, Cell, and Developmental Biology, University of California, Los Angeles, CA 90095 USA

**Keywords:** Cell biology, Computational biology and bioinformatics, Molecular biology, Physiology, Diseases

## Abstract

We recently showed that NOTUM, a liver-secreted Wnt inhibitor, can acutely promote browning of white adipose. We now report studies of chronic overexpression of NOTUM in liver indicating that it protects against diet-induced obesity and improves glucose homeostasis in mice. Adeno-associated virus (AAV) vectors were used to overexpress GFP or mouse *Notum* in the livers of male C57BL/6J mice and the mice were fed an obesifying diet. After 14 weeks of high fat, high sucrose diet feeding, the AAV-Notum mice exhibited decreased obesity and improved glucose tolerance compared to the AAV-GFP mice. Gene expression and immunoblotting analysis of the inguinal fat and brown fat revealed increased expression of beige/brown adipocyte markers in the AAV-Notum group, suggesting enhanced thermogenic capacity by NOTUM. A β3 adrenergic receptor agonist-stimulated lipolysis test suggested increased lipolysis capacity by NOTUM. The levels of collagen and C–C motif chemokine ligand 2 (CCL2) in the epididymal white adipose tissue of the AAV-Notum mice were significantly reduced, suggesting decreased fibrosis and inflammation, respectively. RNA sequencing analysis of inguinal white adipose of 4-week chow diet-fed mice revealed a highly significant enrichment of extracellular matrix (ECM) functional cluster among the down-regulated genes in the AAV-Notum group, suggesting a potential mechanism contributing to improved glucose homeostasis. Our in vitro studies demonstrated that recombinant human NOTUM protein blocked the inhibitory effects of WNT3A on brown adipocyte differentiation. Furthermore, NOTUM attenuated WNT3A’s effects on upregulation of TGF-β signaling and its downstream targets. Overall, our data suggest that NOTUM modulates adipose tissue function by promoting thermogenic capacity and inhibiting fibrosis through inhibition of Wnt signaling.

## Introduction

Mammals possess at least two types of thermogenic adipocytes: classical brown adipocytes and beige adipocytes^1,2^. In rodents and infants, classical brown adipocytes-containing brown adipose tissues (BAT) are located in the interscapular regions and around the kidney, whereas beige adipocytes are an inducible form of thermogenic adipocytes that sporadically reside within white adipose tissue (WAT)^2–4^. Both types of adipocytes possess abundant cristae-dense mitochondria that express uncoupling protein 1 (UCP1) and multilocular lipid droplets^5^. Brown adipose tissue activity was detected in a majority of human adults after cold exposure^6,7^. After a 5-h tolerable cold exposure, lean subjects were shown to have more than 2.5-fold higher BAT activity compared to the obese subjects^8^ as measured by ^18^F-labeled fluorodeoxyglucose positron emission tomography/computerized tomography. These data suggest that targeting thermogenesis can be a promising approach for treating obesity.

We recently developed a system**s-**genetics approach for the discovery of novel endocrine factors^9^. One of the factors predicted to underlie a liver-adipose axis was NOTUM, a carboxylesterase that inactivates WNTs by cleaving the palmitoleate moiety essential for Frizzled receptor binding and activation^10–12^. Wnt pathways are known to regulate adipogenesis^13–15^. Canonical Wnt/β-catenin signaling pathway inhibits adipocyte differentiation by repressing the expression of pro-adipogenic transcription factors peroxisome proliferator-activated receptor gamma (PPARγ) and CCAAT-enhancer-binding protein alpha (C/EBPα)^14,15^. Activation of Wnt signaling blocks brown adipogenesis *in vitro*^16^. Wnt family member 10a (*Wnt10a*) and Wnt family member 10b (*Wnt10b*) are expressed in mouse BAT^16^. Overexpression of *Wnt10b* in BAT under the control of a UCP1 promoter leads to greatly reduced expression of PPARγ coactivator 1 alpha (PGC1α) and UCP1, and lack of functional BAT^16^. Conversely, inhibition of Wnt signaling pathway has been shown to enhance browning of mouse primary white adipocytes^17^.

We previously showed that *Notum* expression in liver was strongly associated with the expression of mitochondrial genes, including *Ucp1*, in WAT^9^. Purified NOTUM induced a thermogenic program in white and brown pre-adipocyte cell lines and primary pre-adipocytes in vitro, and overexpression of NOTUM using adenoviral delivery increased *Ucp1* expression in both white and brown adipose tissues and enabled the mice to maintain a core body temperature during cold exposure^9^. Consistent with these data, aged male liver-specific *Notum* knockout mice on a chow diet exhibited increased obesity and impaired glucose homeostasis compared to the wild-type mice^18^, suggesting an anti-obesity function of liver-derived NOTUM.

We now report stidies of long-term effects of NOTUM overexpression on obesity and glucose homeostasis using adeno-associated virus (AAV)-mediated overexpression in liver. NOTUM increased the expression of thermogenic genes in WAT and BAT, resulting in reduced weight gain on a high fat diet and improved systemic glucose homeostasis. In addition, it inhibited the expression of extracellular matrix genes and decreased collagen content in WAT. Studies in a brown pre-adipocyte cell line showed that recombinant NOTUM protein blocked the inhibitory effects of WNT3A on brown adipocyte differentiation. Furthermore, NOTUM attenuated the effects of WNT3A on upregulation of TGF-β signaling and its downstream target collagen genes.

## Results

### Hepatic NOTUM overexpression protected against diet-induced obesity in mice

**Figure 1:**
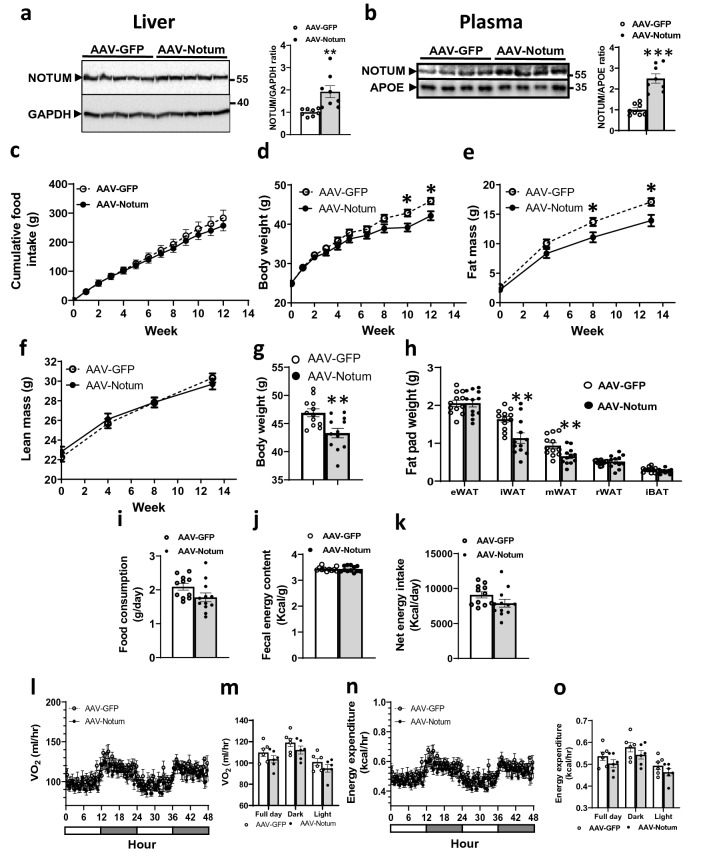
NOTUM overexpression protects against diet-induced obesity in mice. (**a**–**k**) Male C57BL/6J mice were infected with AAV-GFP or AAV-Notum (n = 12 per group) and fed a HF/HS diet for 14 weeks to induce obesity. (**a**) Liver NOTUM and GAPDH protein levels as determined by immunoblotting (n = 8/group). The images shown for NOTUM and GAPDH were from different parts of the same blot. Liver NOTUM/GAPDH ratios are shown in the right panel. (**b**) Plasma NOTUM and APOE levels as determined by immunoblotting. n = 8/group. The images shown for NOTUM and APOE were from different parts of same blot. The plasma NOTUM/APOE ratios are shown in the right panel. (**c**) Average cumulative food intake per mouse during the first 12 weeks of diet feeding is shown. Body weight (**d**), fat mass (**e**), and lean mass (**f**) of mice during the diet feeding period are shown. n = 12/group. At euthanasia (14 weeks), body weight (**g**), and weights of eWAT, iWAT, mWAT, rWAT, and iBAT (**h**) were measured. n = 12/group. (**i**–**k**), n = 11 to 12/group: After five-week feeding of the HF/HS diet, mice were individually housed for 48 h for measurements of food consumption (**i**). Fecal samples were collected for determination of fecal energy content (**j**). (**k**) Net energy intake was then calculated (see “Material and methods” section). (**l**–**o**) In an independent experiment, AAV-GFP and AAV-Notum infected mice (n = 6/group) were fed a HF/HS diet for 6 weeks before the mice were individually housed in metabolic cages for 3 days for measurement of energy expenditure by indirect calorimetry. Oxygen consumption rate, (**l**) and (**m**), and energy expenditure rate (**n**) and (**o**) are shown. Symbols: *: *p* < 0.05, **: *p* < 0.01, ***: *p* < 0.001, AAV-GFP versus AAV-Notum as determined by Student’s t-test.

Eight-week-old male C57BL/6J mice were infected with either AAV encoding green fluorescence protein (GFP, AAV-GFP) or AAV encoding mouse *Notum* (AAV-Notum) to drive expression of GFP or *Notum* in the liver using a human thyroxine binding globulin (TBG) promoter. Hepatic and plasma NOTUM protein levels from the AAV-Notum treated mice were increased approximately 2- and 2.5-fold, respectively, compared to the AAV-GFP group (Fig. 1a,b). The mice were fed a high fat/high sucrose diet for 14 weeks to promote obesity. AAV-Notum treated mice did not show significant differences in food intake during the course of the study compared to the AAV-GFP treated mice (Fig. 1c). However, we observed significantly decreased body weight in the AAV-Notum mice at 10 and 12 weeks of the diet feeding (Fig. 1d). Total fat mass was significantly decreased in the AAV-Notum mice at 8 and 13 weeks (Fig. 1e), whereas total lean mass was similar between the 2 groups of mice throughout the study period (Fig. 1f). At euthanasia (14-week diet feeding), we observed that the AAV-Notum treated mice had significantly decreased body weight (Fig. 1g) but similar weights of epididymal WAT (eWAT), retroperitoneal WAT (rWAT), and interscapular BAT (iBAT) compared to the AAV-GFP treated mice (Fig. 1h). On the other hand, the weights of inguinal WAT (iWAT) and mesenteric WAT (mWAT) were significantly decreased (Fig. 1h) in AAV-Notum treated mice. These results demonstrate that NOTUM protects against diet-induced obesity and reduces total fat mass in mice.

### AAV-Notum treated mice exhibited similar food intake and energy expenditure compared to the AAV-GFP treated mice

In a separate experiment, food consumption was measured after 5 weeks of diet feeding, before differences in body weight appeared, in singly housed mice. We did not observe significant differences in daily food consumption (Fig. 1i), fecal energy content (Fig. 1j), or net energy intake (Fig. 1k) between the two groups of mice, suggesting similar food intake and intestinal nutrient absorption. In an effort to understand the basis of the reduced adiposity resulting from NOTUM overexpression, we performed whole body indirect calorimetry in metabolic cage. The metabolic cage-derived data were analyzed by performing analysis of covariance (ANCOVA)^19^ using body weight, lean mass, or fat mass of the mice, separately, as a covariate. After six weeks of HF/HS diet feeding, we did not observe significant differences in oxygen consumption (Fig. 1l,m), energy expenditure (Fig. 1n,o), respiratory exchange ratio (data not shown), or physical activity (data not shown) between the two groups of mice using any of the body weight, lean or fat mass as a covariate (data not shown).

### AAV-Notum treated mice maintained on a HF/HS diet exhibited altered plasma lipoprotein profiles, decreased insulin levels, and similar liver traits compared to AAV-GFP mice

**Figure 2:**
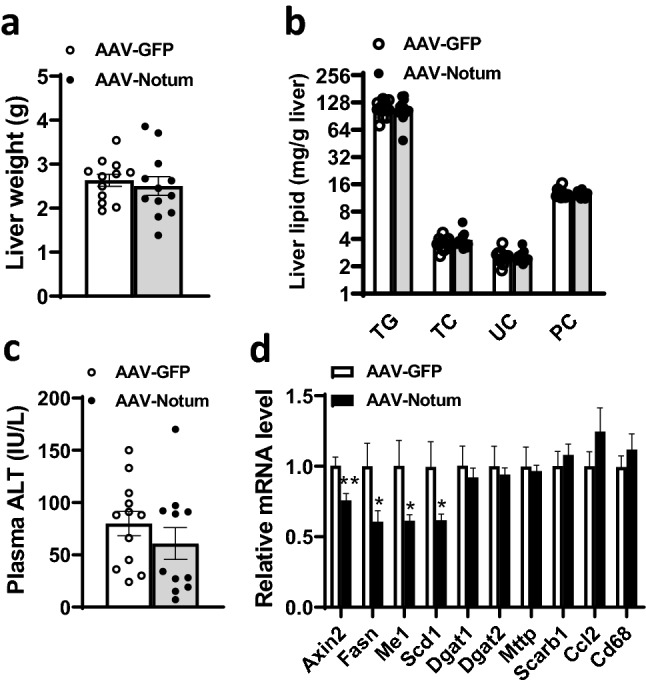
NOTUM overexpression did not affect liver weight, hepatic lipid content, or induce liver injury. Liver and plasma samples from mice described in Fig. 1a–j (n = 11–12 per group) were examined for (**a**) liver weight, (**b**) liver lipid content including triglyceride (TG), total cholesterol (TC), unesterified cholesterol (UC), and phosphatidylcholine (PC), (**c**) plasma ALT levels, and (**d**) liver gene expression by qPCR. Symbols are the same as Fig. 1.

At 14 weeks, fasting plasma glucose levels of the AAV-Notum mice were similar to those of the AAV-GFP mice (Table 1). However, fasting plasma insulin levels were significantly lower in the AAV-Notum mice (Table 1). Fasting plasma total-, HDL-, and VLDL/IDL/LDL-cholesterol levels were significantly decreased in the AAV-Notum mice compared to those of the AAV-GFP mice (Table 1) as well. Notably, we observed a significant increase in plasma free fatty acid but similar plasma glycerol levels in the AAV-Notum group compared to the AAV-GFP group (Table 1). On the other hand, liver weight (Fig. 2a), liver triglycerides, phosphatidylcholine, total cholesterol, and unesterified cholesterol levels (Fig. 2b) were similar between the two groups. Plasma levels of alanine aminotransferase (ALT), a marker of liver injury, were similar between the 2 groups as well (Fig. 2c). Liver gene expression analysis by qPCR revealed decreased mRNA levels of Axin2, a Wnt target gene^20^, in AAV-Notum group compared to AAV-GFP group (Fig. 2d). The mRNA levels of genes involved in lipogenesis, including fatty acid synthase (*Fasn*), malic enzyme 1 (*Me1*), and stearoyl-Coenzyme A desaturase 1 (*Scd1*), were significantly decreased in AAV-Notum group compared to controls (Fig. 2d). However, the mRNA levels of genes involved in triglyceride synthesis [diacylglycerol O-acyltransferase 1 (*Dgat1*) and diacylglycerol O-acyltransferase 2 (*Dgat2*)], VLDL assembly (microsomal triglyceride transfer protein, *Mttp*), HDL cholesterol uptake(scavenger receptor class B member 1, *Scarb1*), and inflammation [*Ccl2* and cluster of differentiation 68 (*Cd68*)] were similar between the two groups (Fig. 2d). These data suggest that NOTUM overexpression did not induce adverse effects in the liver.

**Table 1:** Plasma lipids, lipoproteins, glucose, and insulin levels of mice that received AAV-GFP or AAV-Notum and fed a HF/HS diet for 14 weeks.

Parameter	Triglyceride	Total Chol	HDL Chol	VLDL/IDL/LDL Chol	FFA	Glycerol	Glucose	Insulin
Unit	mg/dL	mg/dL	mg/dL	mg/dL	mg/dL	mg/dL	mg/dL	pg/ml
AAV-GFP (n = 12)	17.3 ± 1.1	258 ± 7	194 ± 4	64 ± 4	27 ± 1	41 ± 4	269 ± 12	4130 ± 403
AAV-Notum (n = 12)	15.0 ± 1.2	227 ± 6	180 ± 3	47 ± 3	33 ± 1	39 ± 3	253 ± 8	2838 ± 375
*p* value	0.18	0.004	0.01	0.003	0.0001	0.67	0.28	0.03

Values shown are means ± standard errors.

*Chol.* Cholesterol, *FFA* Free fatty acids.

### AAV-Notum treated mice exhibited improved glucose tolerance

**Figure 3:**
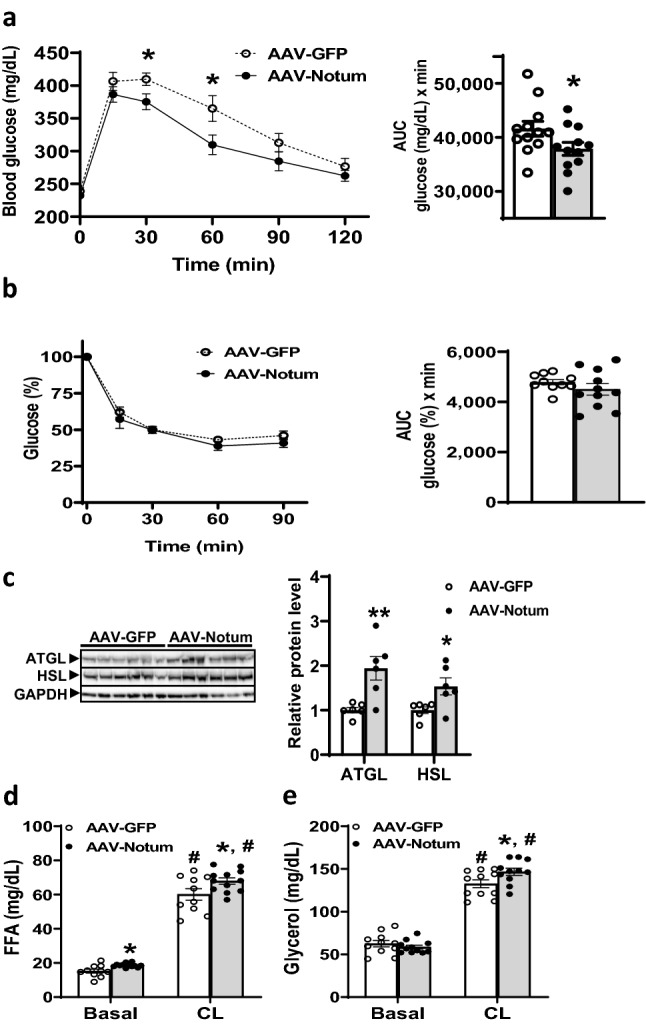
NOTUM overexpression improves glucose tolerance and increases lipolysis capacity. AAV-GFP and AAV-Notum infected mice (n = 11 to 12 mice per group) were maintained on a HF/HS diet for 10 to 12 weeks before the following experiments were performed. (**a**) IPGTT was performed as described in “Material and methods” section. Blood glucose levels, right, and glucose AUC, left, are shown. (**b**) ITT was performed as described in “Material and methods”. Blood glucose levels, left, and glucose AUC, right, are shown. (**c**) ATGL and HSL protein levels in iWAT were determined by immunoblotting (n = 6 per group), left. Relative protein levels of ATGL and HSL, as normalized by GAPDH levels, were shown in right. Images of ATGL, HSL, and GAPDH were obtained from different parts of the same blot. (**d**, **e**) β3 adrenergic receptor agonist stimulated lipolysis was performed as described in “Material and methods” section. Plasma free fatty acid (FFA, **d**) and glycerol (**e**) levels at time 0 (basal) and 30 min after injection of CL316,243 (CL) are shown (n = 10–12 per group). Symbols: *: *p* < 0.05, **: *p* < 0.01, AAV-GFP versus AAV-Notum as determined by Student’s t-test. For (**c**) and (**d**), #: *p* < 0.05 versus time 0 (basal) of the same AAV group.

Intraperitoneal glucose tolerance test (IPGTT) was performed on AAV-Notum and AAV-GFP mice after 12 weeks of HF/HS diet feeding. We observed significantly lower blood glucose levels at 30 and 60 min post glucose injection in the AAV-Notum mice compared to those of the AAV-GFP mice (Fig. 3a). Glucose area under the curve (AUC) values for the AAV-Notum mice were also significantly lower (Fig. 3a), indicating improved glucose tolerance.

Insulin tolerance test (ITT) was performed to evaluate insulin sensitivity after 11 weeks of HF/HS diet feeding. We observed that AAV-Notum mice exhibited lower levels of blood glucose at time 0 (251 ± 9 vs. 276 ± 8 mg/dL, *p* = 0.05) as compared to the AAV-GFP mice. Therefore, glucose values at all time points (0, 15, 30, 60, and 90 min) were normalized to values at time 0 of each mouse for data analysis. We observed similar glucose lowering responses to insulin in AAV-GFP and AAV-Notum mice (Fig. 3b), suggesting similar insulin sensitivity between these two groups of mice when exogenous insulin was administered based on body weight.

### AAV-Notum treated mice demonstrated increased lipolysis capacity induced by β3 adrenergic receptor agonist treatment

We observed that the protein levels of adipose triglyceride lipase (ATGL) and hormone sensitive lipase (HSL), enzymes responsible for lipolysis, were increased by 94% and 54%, respectively, in the iWATs of AAV-Notum group (Fig. 3c), suggesting these might contribute to the significantly increased plasma FFA levels observed in the AAV-Notum mice (Table 1). Furthermore, we performed β3 adrenergic receptor agonist-stimulated lipolysis experiments to determine whether the increased fasting free fatty acid levels observed in the AAV-Notum mice (Table 1) were due, in part, to increased WAT lipolysis. We observed about a threefold increase in plasma FFA levels (Fig. 3d) and twofold increase in plasma glycerol levels (Fig. 3e) in both groups of mice 30 min after receiving CL316,243 injection compared to time 0. As shown in Fig. 3d, compared to the AAV-GFP mice, AAV-Notum mice exhibited significantly higher FFA levels at time 0 (basal) and 30 min post CL316,243 injection. While there were no significant differences in plasma glycerol levels at time 0 (Fig. 3e), AAV-Notum mice exhibited significantly higher glycerol levels compared to the controls at 30 min (Fig. 3e). These data suggest that WATs from the AAV-Notum mice possess increased lipolysis capacity in response to β3 adrenergic receptor agonist stimulation compared to the controls.

### NOTUM promoted thermogenic capacity of WAT and BAT and decreased inflammation and fibrosis in WAT

**Figure 4:**
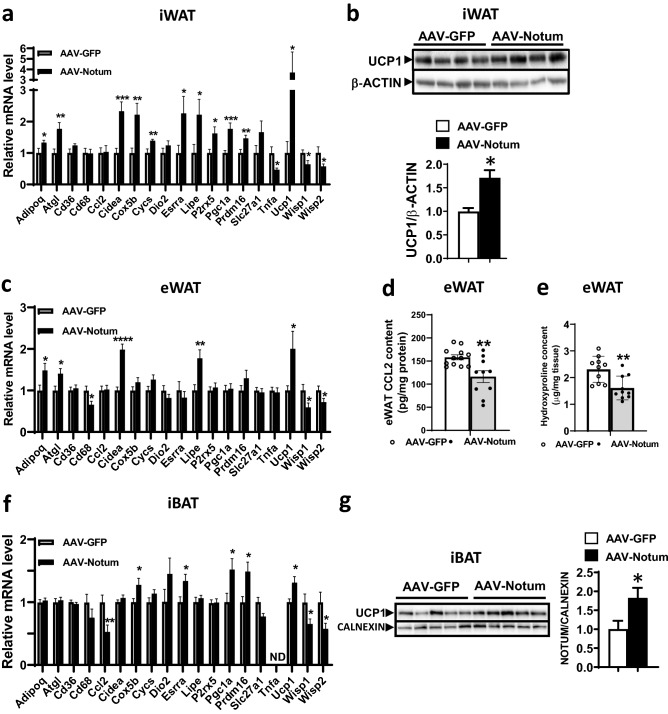
NOTUM overexpression promotes thermogenic capacity in WATs and iBAT and decreases adipose tissue inflammation and fibrosis. IWAT, eWAT, and iBAT samples from the same mice described in Fig. 1a–i were used for the following assays. (**a**) Gene expression analysis by qPCR of iWATs are shown. n = 9–12/group. (**b**) Protein levels of UCP1 and β-ACTIN in the iWAT were determined by immunoblotting (top) and quantification of the UCP1 protein normalized by the β-ACTIN protein is shown at the bottom. n = 4. Images of UCP1 and β-ACTIN were obtained from different parts of same blot. Gene expression analysis by qPCR, (**c**), CCL2 protein content, (**d**), and hydroxyproline content, (**e**), of eWAT are shown. Gene expression analysis (n = 10–12), (**f**), and UCP1 and β-ACTIN protein levels as determined by immunoblotting, (**g**), (n = 5) of iBAT are shown. Images of UCP1 and β-ACTIN were obtained from different parts of same blot. Symbols: #: *p* ≤ 0.09, *: *p* < 0.05, **: *p* < 0.01, ***: *p* < 0.001, AAV-GFP versus AAV-Notum, as determined by Student’s t-test.

After 14-week HF/HS diet feeding, gene expression analysis of iWAT of the AAV-Notum treated mice revealed significantly decreased mRNA levels of Wnt target genes, WNT1-inducible-signaling pathway protein 1 (*Wisp1*)^21^ and WNT1-inducible-signaling pathway protein 2 (*Wisp2*)^21^, and increased expression of thermogenic/beige adipocyte-enriched genes, including cell death inducing DFFA like effector a (*Cidea*), purinergic receptor P2X 5 (*P2rx5*), *Pgc1a*, PR/SET domain 16 (*Prdm16*), and *Ucp1* compared to those of the AAV-GFP mice (Fig. 4a). Furthermore, UCP1 protein levels were significantly higher in the iWAT of AAV-Notum mice compared to those of the control mice (Fig. 4b). The expression level of the transcription factor estrogen-related receptor α (ERR encoded by *Esrra*), a target gene of PGC1α^22,23^, was significantly increased in the iWAT of AAV-Notum group compared to the AAV-GFP group (Fig. 4a). ERRα is known to cooperate with PGC1α to drive the expression of genes involved in mitochondrial biogenesis and oxidative phosphorylation (OXPHOS), such as cytochrome C oxidase subunit 5b (*Cox5b*) and cytochrome C (*Cycs*)^22,23^. As expected, we observed significantly increased expression levels of *Cox5b* and *Cycs* in the AAV-Notum treated group (Fig. 4a). In addition, expression levels of adiponectin (*adipoq*), a beneficial adipokine that sensitizes the body to insulin^24^, were significantly higher in the AAV-Notum mice (Fig. 4a), whereas the mRNA levels of tumor necrosis factor α (*Tnfα*), a cytokine that promotes insulin resistance^25^, were significantly lower in the AAV-Notum mice (Fig. 4a). The mRNA levels of lipolysis genes, *Atgl* and *Lipe*, were significantly increased in AAV-Notum group (Fig. 4a), whereas those of fatty acid transporters, cluster of differentiation 36 (*Cd36*) and solute carrier family 27 member 1 (*Slc27a1*), were similar (Fig. 4a). There were no significant differences in the expression of *Cd68* (a macrophage marker) or *Ccl2* (a chemokine) between the 2 groups (Fig. 4a). Similarly, in eWAT of the AAV-Notum treated mice, we observed significantly increased mRNA levels of *Adipoq*, *Cidea*, *Ucp1*, *Atgl*, and *Lipe*, and decreased mRNA levels of *Cd68*, *Wisp1* and *Wisp2* (Fig. 4c). In addition, C–C motif chemokine ligand 2 (CCL2) protein levels (a principal chemokine involved in macrophage infiltration, adipose inflammation and insulin resistance^26^) were significantly lower in the eWAT of the AAV-Notum treated mice (Fig. 4d). The collagen content of eWAT was determined by measuring hydroxyproline levels. There was a significant decrease in hydroxyproline content in the eWAT of AAV-Notum group (Fig. 4e), suggesting suppression of fibrosis by NOTUM in adipose tissue. These data demonstrated that NOTUM overexpression led to increased beiging, increased expression of OXPHOS genes, and decreased inflammation and fibrosis in WAT.

Gene expression analysis of iBAT revealed that the mRNA levels of Wnt target genes, *Wisp1* and *Wisp2*, and inflammatory gene, *Ccl2*, were decreased in AAV-Notum group (Fig. 4f). In addition, genes associated with thermogenic function, including *Ucp1*, *Prdm16*, and *Pgc1a,* and genes associated with mitochondrial biogenesis and OXPHOS, *Cox5b* and *Esrra*, were increased in the iBAT of AAV-Notum mice compared to the AAV-GFP mice (Fig. 4f). On the other hand, transcript levels of lipolysis genes, *Atgl* and *Lipe*, were similar between the two groups (Fig. 4f). Lastly, UCP1 protein levels in the iBATs of AAV-Notum mice were significantly increased by 2.6-fold compared to controls (Fig. 4g).

### NOTUM overexpression decreased adipocyte hypertrophy, increased mitochondrial DNA content in WAT and preserved brown adipocyte morphology in BAT

**Figure 5:**
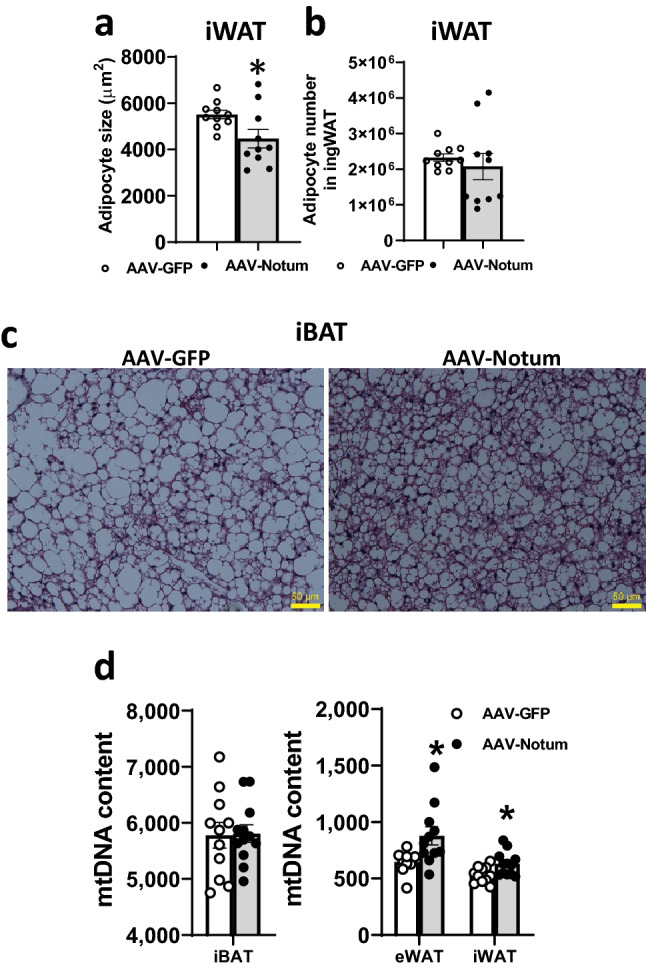
NOTUM overexpression decreases adipocyte hypertrophy in iWAT, preserves BAT morphology, and increases mitochondrial DNA content in WATs. Tissue samples were from mice described in Fig. 1a–j. Average adipocyte size, (**a**) and total adipocyte number, (**b**) of iWAT were determined as described in the “Material and methods” section. n = 10 per group. (**c**) Representative H&E stained histological sections of iBAT are shown. (**d**) Mitochondrial DNA (mtDNA) contents of eWAT, iWAT, and iBAT are shown (n = 9–12). Symbols:*: *p* < 0.05, AAV-GFP versus AAV-Notum, as determined by Student’s t-test.

The average adipocyte size of iWAT from the AAV-Notum mice was significantly reduced compared to those of the AAV-GFP mice (Fig. 5a), demonstrating decreased adipocyte hypertrophy in the former. While there were no significant differences in total adipocyte number in the iWAT between the 2 groups (Fig. 5b), there was a distinct difference in morphology of the iBAT. Histological examination of iBAT from the AAV-Notum mice showed an abundance of typical brown adipocytes with the presence of multiple small lipid droplets in each cell (Fig. 5c). The iBAT of AAV-GFP mice, in contrast, contained many “white adipocyte”-like cells with large lipid droplets (Fig. 5c), suggesting NOTUM overexpression preserved BAT morphology.

We determined mitochondrial DNA (mtDNA) content, normalized by nuclear DNA, of various fat pads using quantitative polymerase chain reaction (qPCR). The eWATs and iWATs of AAV-Notum mice exhibited significantly increased mtDNA content, by 36% and 18%, respectively, compared to those of the AAV-GFP mice (Fig. 5d). In contrast, the mtDNA contents in iBATs of the two groups of mice were similar (Fig. 5d).

### Short-term hepatic NOTUM overexpression led to decreased adiposity and substantial changes in gene expression in WAT

**Figure 6:**
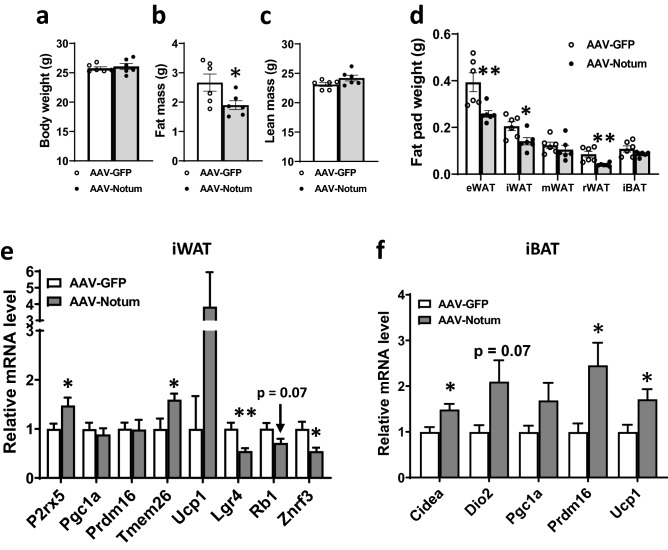
NOTUM overexpression decreases adiposity in chow diet-fed mice. Male C57BL/6J mice were infected with AAV-GFP or AAV-Notum (n = 6 per group) and maintained on a low fat chow diet for 4 weeks before tissue collection. Body weight (**a**), fat mass (**b**), lean mass (**c**), and fat pad weights (**d**) are shown. Gene expression analysis by qPCR of iWAT (**e**), n = 6, and iBAT (**f**), n = 4–5, are shown. Symbols are the same as shown in Fig. 1.

To elucidate the underlying mechanism(s) by which NOTUM prevents obesity, we performed a 4-week, short-term NOTUM overexpression study in mice maintained on a low fat chow diet. We observed a significant decrease in fat mass, but no significant differences in body weight or lean mass in the AAV-Notum group, compared to the AAV-GFP group (Fig. 6a–c). Furthermore, the eWAT, iWAT, and rWAT weights of AAV-Notum mice were significantly decreased, whereas mWAT and iBAT weights were similar between the 2 groups (Fig. 6d). No significant changes in plasma triglycerides, lipoproteins, glycerol, glucose, or insulin levels were observed in the AAV-Notum treated mice (Table 2). However, plasma FFA levels were significantly increased in the AAV-Notum mice (Table 2), suggesting increased WAT lipolysis. Gene expression analysis of iWAT by qPCR revealed significant increases in the expression of beige adipocyte markers, including *P2rx5* and transmembrane protein 26 (*Tmem26*) (Fig. 6e), and significant decreases in the expression of genes that inhibit beiging [leucine rich repeat containing G protein-coupled receptor 4 (*Lgr4*)^27^ and RB transcriptional corepressor 1 (*Rb1*)^28,29^] in the AAV-Notum group compared to the AAV-GFP group. We also observed that NOTUM overexpression was associated with a significant decrease in zinc and ring finger 3 (*Znrf3*), a Wnt/β-catenin target gene and negative feedback regulator of Wnt signaling^30^. These data suggest that NOTUM suppresses Wnt/β-catenin signaling and promotes beige adipogenesis in iWAT. In addition, we observed significantly higher expression of thermogenic genes, including *Cidea*, *Prdm16*, and *Ucp1*, in the iBAT of AAV-Notum treated mice (Fig. 6f).

**Table 2:** Plasma lipids, lipoproteins, glucose, and insulin levels of mice that received AAV-GFP or AAV-Notum and fed a chow diet for 4 weeks.

Parameter	Triglyceride	Total Chol	HDL Chol	VLDL/IDL/LDL Chol	FFA	Glycerol	Glucose	Insulin
Unit	mg/dL	mg/dL	mg/dL	mg/dL	mg/dL	mg/dL	mg/dL	pg/ml
AAV-GFP (n = 6)	36 ± 2	100 ± 3	80 ± 2	20 ± 1	45 ± 3	45 ± 2	186 ± 4	619 ± 134
AAV-Notum (n = 6)	38 ± 3	98 ± 3	78 ± 3	20 ± 2	66 ± 1	47 ± 2	203 ± 9	549 ± 29
*p* value	0.64	0.67	0.67	0.86	0.0002	0.68	0.13	0.62

Values shown are means ± standard errors.

*Chol.* Cholesterol, *FFA* Free fatty acids.

**Figure 7:**
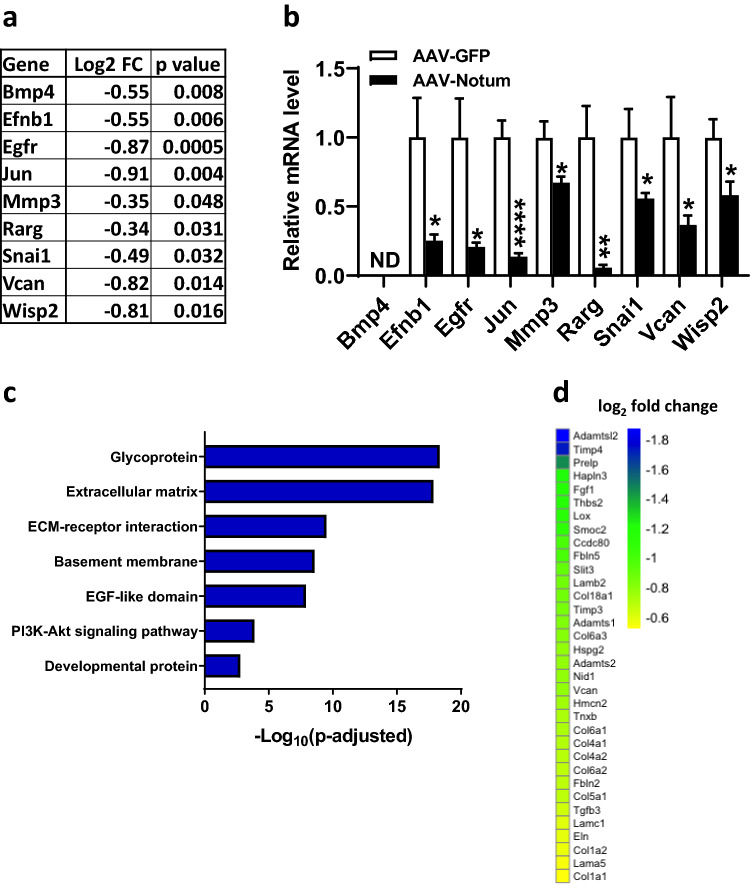
NOTUM overexpression leads to substantial changes in global gene expression in iWAT of chow diet-fed mice. RNA-seq analysis were performed on iWAT samples collected from the same chow diet fed mice as described in Fig. 6. (**a**) Wnt target genes that were differentially expressed between the AAV-Notum and AAV-GFP groups were shown. (**b**) qPCR data of the same Wnt target genes described in (**a**) are shown. (**c**) Repressed functional clusters in the iWAT of AAV-Notum treated mice compared to the AAV-GFP treated mice are shown. (**d**) Heat map of log_2_ fold changes of ECM cluster transcripts in the iWAT of AAV-Notum treated mice compared to those of the AAV-GFP treated mice are shown. Symbols: *: *p* < 0.05, **: *p* < 0.01, ****: *p* < 0.0001, AAV-GFP versus AAV-Notum, as determined by Student’s t-test. *ECM* Extracellular matrix, *EGF* Epidermal growth factor, *FC* Fold change, *ND* Not detectable.

We then performed RNA sequencing (RNA-seq) analysis on iWAT of AAV-GFP and AAV-Notum treated mice to examine NOTUM’s effect on global gene expression. Based on RNA-seq data, we found that 9 out of the 55 (16%) expressed Wnt target genes^20,31–66^ (Supplemental Table 1) were significantly decreased in the iWAT of AAV-Notum mice compared to AAV-GFP mice (Fig. 7a). These 9 genes are bone morphogenetic protein 4 (*Bmp4*)*,* ephrin-B1 (*Efnb1*), epidermal growth factor receptor (*Egfr*)*,* Jun proto-oncogene (*Jun*), matrix metalloproteinase 3 (*Mmp3*), retinoic acid receptor gamma (*Rarg*), Snail family transcriptional repressor 1 (*Snai*), versican (*Vcan*), and *Wisp2* (Fig. 7a). Using qPCR, we were able to confirm decreased expression for 8 of the 9 Wnt target genes (Fig. 7b). However, our qPCR method was not sensitive enough to detect *Bmp4* transcripts in iWAT. These data suggested that Notum negatively impacted expression of some Wnt target genes in iWAT, probably through inhibition of Wnt signaling. Globally, we observed that 78 genes were up-regulated and 334 genes were down-regulated (adjusted p value < 0.05) in the iWAT of AAV-Notum mice. These differentially expressed genes were selected for gene ontology analysis. The only enriched cluster associated with up-regulated genes was “Cellular response to interferon-beta” (Supplemental Table 2). There were 7 major enriched functional clusters (Fig. 7c and Supplemental Table 3) associated with down-regulated genes, including glycoprotein, extracellular matrix (ECM), ECM-receptor interaction, basement membrane, epidermal growth factor-like domain, PI3K-Akt signaling pathway and developmental protein (Fig. 7c). There were 34 down regulated genes in the ECM cluster that includes 6 collagen genes: *Col1a1*, *Col4a1*, *Col4a2*, *Col5a1*, *Col6a1*, *Col6a2*, *Col6a3*, and *Col18a1* (Fig. 7d). The Wnt signaling pathways have previously been shown to promote fibrosis^67^. Our data suggest that NOTUM inhibits Wnt signaling, leading to down-regulation of ECM genes such as collagens that are constituents of fibrosis.

### NOTUM attenuated WNT3A’s blocking effects on brown adipocyte differentiation and upregulating TGF-β signaling in vitro

**Figure 8:**
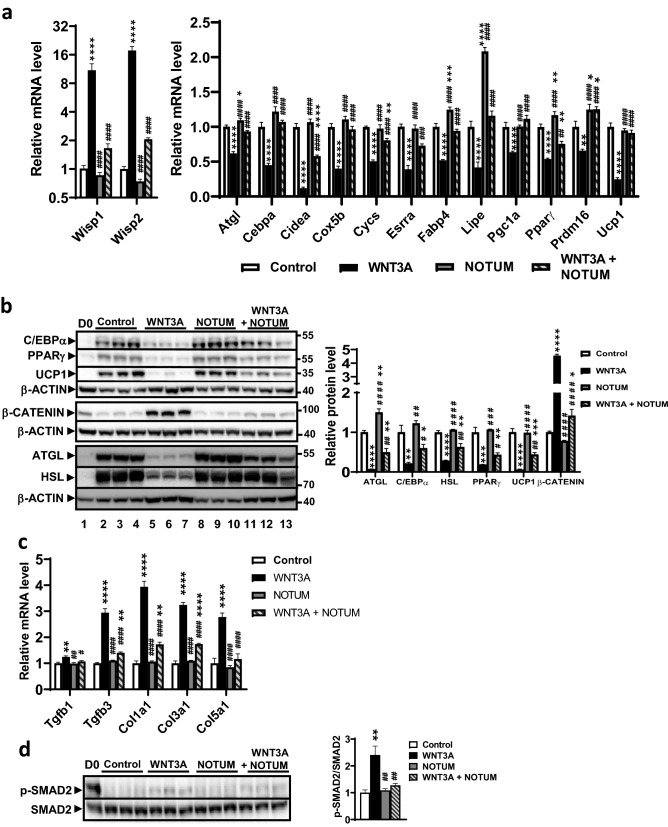
NOTUM abolishes WNT3A’s inhibitory effect on brown adipocyte differentiation in vitro. Confluent pre-BAT cells were treated for 7 days with media promoting brown adipocyte differentiation that contain BSA (1 μg/ml, control), 10 ng/ml human WNT3A, 300 ng/ml human NOTUM, or 10 ng/ml human WNT3A and human 300 ng/ml NOTUM for 7 days before gene expression analysis and immunoblotting. The mRNA (**a**) (n = 4–6/group) and protein (**b**) (n = 3/group) levels of genes involved in brown adipocyte differentiation and Wnt signaling are shown. For (**b**) one sample from baseline (Day 0, D0) was included as reference. For (**b**), The images shown in the top 4 panels were obtained from the same blot: C/EBPα, PPARγ, and β-ACTIN images were obtained from the the top part of the same blot after stripping and re-probing, whereas image of UCP1 was obtained from the bottom part of the same blot. The images shown in the middle 2 panels of (**b**), b-CATENIN and β-ACTIN, were obtained from top and bottom portions of the same blot, respectively. The images shown in the bottom 3 panels of (**b**), ATGL, HSL, and β-ACTIN, were obtained from the same blot. HSL was obtained from the top portion of the blot. ATGL and β-ACTIN were obtained from the bottom portion of the blot after stripping and re-probing. Individual proteins to β-ACTIN ratios are shown in the right panel. (**c**) The mRNA levels of genes involved in TGF-β signaling and collagen deposition are shown (n = 4–5/group). (**d**) Activation of TGF-β signaling in pre-BAT at Day 7 as determined by p-SMAD2 to SMAD2 ratio is shown (n = 3/group). One sample at day 0 (D0) was shown as reference. The same blot was first probed with the anti p-SMAD2 antibodies to obtain the p-SMAD2 image, the blot was then stripped and re-probed with the anti SMAD2 antibody to obtain the SMAD2 image. The ratios of p-SMAD2 to SMAD2 are shown in the right panel. Symbols: *: *p* < 0.05, **: *p* < 0.01, ***: *p* < 0.001, ****: *p* < 0.0001, as compared to control group. #: *p* < 0.05, ##: *p* < 0.01, ###: *p* < 0.001, ####: *p* < 0.0001, as compared to WNT3A group. The Holm–Sidak post hoc analysis was used for between-group comparisons after statistical significance was established by ANOVA (see “Material and methods” section).

Wnt signaling pathway is known to inhibit white and brown adipocyte differentiation^14–16^. We investigated whether NOTUM can block the inhibitory effects of Wnt signaling on brown adipocyte differentiation in vitro. Brown pre-adipocytes undergoing differentiation were treated with recombinant human WNT3A, NOTUM, or both proteins for 7 days to determine whether NOTUM can alleviate the previously reported inhibitory effects of WNT3A on brown adipocyte differentiation^16^. In the WNT3A treated cells as compared to the control cells, we observed an 11-fold increase in the mRNA level of *Wisp1*, a Wnt target gene which is known to inhibit adipocyte differentiation^21,68^ (Fig. 8a). NOTUM treatment alone did not significantly change *Wisp1* mRNA level whereas WNT3A + NOTUM group exhibited an 85% decrease in *Wisp1* mRNA level compared to WNT3A group (Fig. 8a), suggesting that NOTUM blocked WNT3A signaling. Similar findings were observed in the expression of another Wnt target gene, *Wisp2*^21^. Thus, we observed an 18-fold increase in the mRNA level of *Wisp2* in the WNT3A treatment group compared to the control group (Fig. 8a). NOTUM treatment alone significantly decreased *Wisp2* mRNA level by 26% whereas WNT3A + NOTUM group exhibited an 88% decrease in *Wisp2* mRNA level compared to WNT3A group (Fig. 8a).

WNT3A treatment significantly decreased the mRNA levels of genes involved in brown adipocyte differentiation and function, including *Atgl*, *Cebpa* , *Cidea*, *Cox5b*, *Cycs*, *Esrra*, fatty acid binding protein 4 (*Fabp4*)*, Lipe, Pgc1α*, *Pparγ, Prdm16* and *Ucp1*, by 34% to 93%, compared to control group (Fig. 8a), demonstrating WNT3A inhibited brown adipocyte differentiation. NOTUM treatment by itself significantly increased the mRNA levels of *Atgl*, *Cebpa*, *Fabp4*, *Lipe*, *Pparγ*, and *Prdm16* by 10% to 105%, compared to the controls (Fig. 8a). Importantly, WNT3A + NOTUM group exhibited significant increases in mRNA levels of *Atgl*, *Cebpa*, *Cidea, Cox5b*, *Cycs*, *Esrra*, *Fabp4, lipe, Pgc1a, Pparγ, Prdm16*, and *Ucp1* by 1.4-fold to 5.2-fold, compared to WNT3A group (Fig. 8a), demonstrating that NOTUM reversed the inhibitory effects of WNT3A on brown adipocyte differentiation. Immunoblotting analysis revealed substantial increases in the protein levels of ATGL, C/EBPα, HSL, PPARγ, and UCP1 in the control cells at day 7 after initiation of brown adipocyte differentiation as compared to the baseline (day 0) (Fig. 8b), confirming that our experimental conditions promoted brown adipocyte differentiation. We observed that WNT3A treatment significantly increased the protein level of active β-CATENIN, a critical transcription factor downstream of Wnt signaling, by 4.5-fold compared to controls (Fig. 8b). Furthermore, WNT3A significantly decreased the protein levels of ATGL, C/EBPα, HSL, PPARγ, and UCP1, by 79% to 94%, compared to controls (Fig. 8b), suggesting substantial inhibition of brown adipocyte differentiation by WNT3A. NOTUM by itself significantly increased ATGL protein level by 50% compared to controls, but did not significantly change the protein levels of C/EBPα, HSL, PPARγ, UCP1, or active β-CATENIN (Fig. 8b). However, WNT3A + NOTUM group exhibited significant increases in the protein levels of ATGL, C/EBPα, HSL, PPARγ, and UCP1, by 2.3-fold to 7.7-fold, and a decrease in active β-CATENIN (by 83%) compared to WNT3A alone (Fig. 8b), demonstrating that NOTUM inhibited the activation of β-CATENIN by WNT3A and increased brown adipocyte differentiation compared to WNT3A group.

WNT3A has been shown to increase the expression of transforming growth factor-β (TGF-β), leading to increased TGF-β signaling through mothers against decapentaplegic homolog 2 (SMAD2) in mouse fibroblasts^69^. We investigated whether NOTUM can block the upregulation of TGF-β signaling by WNT3A in differentiating brown pre-adipocytes. In addition, we examined the expression of collagen genes that are induced by TGF-β signaling^70,71^. WNT3A significantly increased the mRNA levels of transforming growth factor beta 1 (*Tgfb1*), transforming growth factor beta 3 (*Tgfb3*), collagen type I alpha 1 chain (*Col1a1*), collagen type III alpha 1 chain (*Col3a1*), and collagen type V alpha 1 chain (*Col5a1*), by 0.24-fold to 3.9-fold, compared to the control group at day 7 after initiation of differentiation (Fig. 8c). NOTUM treatment did not significantly change the expression of any Tgfβ or collagen genes compared to controls (Fig. 8c). On the other hand, WNT3A + NOTUM group exhibited significant decreases in mRNA levels of *Tgfb1*, *Tgfb3*, *Col1a1*, *Col3a1*, and *Col5a1*, by 17% to 58%, compared to the WNT3A group (Fig. 8c). At baseline (day 0), there was a substantial level of TGF-β signaling, as determined by phosphorylation of SMAD2, compared to control cells after 7 days of differentiation (Fig. 8d). At day 7, TGF-β signaling was significantly higher by the treatment of WNT3A (by 2.4-fold) compared to the control (Fig. 8d), whereas NOTUM treatment did not significantly affect TGF-β signaling compared to the control (Fig. 8d). However, WNT3A + NOTUM group exhibited significantly decreased phosphorylation of SMAD2 (by 47%) compared to WNT3A (Fig. 8d), demonstrating that NOTUM blocked the upregulation of TGF-β signaling by WNT3A in differentiating brown pre-adipocytes.

## Discussion

This study demonstrates that hepatic overexpression of NOTUM leads to decreased diet-induced obesity, improved glucose homeostasis, and increased lipolysis capacity in mice. Following AAV-mediated overexpression of NOTUM, we observed increased beiging in WATs and enhanced thermogenic potential in BAT. Moreover, we observed significant increases in mtDNA content, a measurement for mitochondrial abundance^72,73^, in the eWAT and iWAT of AAV-Notum mice, possibly due to increased expression of Esrra, Pgc1α and Prdm16, known regulators of mitochondrial biogenesis^22,23,74–76^. In addition, decreased expression of inflammatory markers was observed in the WATs of AAV-Notum-treated mice. RNA-seq analysis of iWAT collected from chow-fed AAV-GFP and AAV-Notum infected mice 4 weeks after viral transduction revealed a highly significant enrichment of extracellular matrix (ECM) functional cluster among the down-regulated genes in the AAV-Notum group. Our in vitro study demonstrated that recombinant human NOTUM protein blocked the inhibitory effects of WNT3A on brown adipocyte differentiation. Furthermore, NOTUM attenuated WNT3A’s effects on upregulation of TGF-β signaling and its downstream target collagen genes. Therefore, our data suggest that NOTUM influences adipose tissue function by promotion of thermogenic capacity and inhibition of fibrosis through inhibition of Wnt signaling.

Liver has emerged as an important organ that modulates whole body metabolic traits such as obesity and insulin resistance. For example, liver is the main organ that secrets fibroblast growth factor 21 (FGF21)^77^. Liver-specific FGF21 knockout mice have increased insulin resistance and decreased brown adipose tissue–mediated glucose disposal^77^. In addition, AAV-mediated liver-specific overexpression of FGF21 leads to marked reductions in body weight, adipose tissue hypertrophy and inflammation, and insulin resistance^78^. Another recent study shows that obesity in mice is associated with increased synthesis and secretion of dipeptidyl peptidase 4 (DPP4), which acts with plasma factor Xa to inflame adipose tissue macrophages^79^. Knocking down the expression of DPP4 in hepatocytes through AAV-encoded short hairpin RNA against DPP4 suppresses inflammation of visceral adipose tissue and insulin resistance in obese mice^79^. Our findings demonstrate NOTUM as a novel liver secreted “endocrine” factor that modulates obesity and glucose homeostasis traits by influencing adipose tissue thermogenic capacity and fibrosis.

Wnt pathways are known to regulate adipogenesis^13–15^. Activation of Wnt signaling blocks brown adipocyte differentiation^16^, whereas Wnt inhibition enhances browning of mouse primary white adipocytes in vitro^17^. Overexpression of *Wnt10b* in BAT under the control of a UCP1 promoter in mice leads to conversion of brown adipocytes into white adipocytes, and greatly diminished expression of *Pgc1a* and *Ucp1*, and genes involved in mitochondrial biogenesis and metabolism^16^. Wnt signaling is thought to suppress *Pgc1a* transcription either directly via a β-catenin-lymphoid enhancer binding factor 1(LEF1) complex, or through interference with transcriptional activity of known *Pgc1a* regulators such as activating transcription factor 2 (ATF2), cAMP-response element binding protein (CREB), and forkhead box protein O1 (FOXO1)^16^. Previously, we demonstrated that recombinant NOTUM protein promoted beige and brown adipocyte differentiation *in vitro*^9^. We also showed that acute overexpression of NOTUM in liver, through adenovirus mediated gene delivery, significantly increased thermogenic capacity in WAT and BAT, and made the mice more tolerant to cold^9^. Our current findings show that long-term NOTUM overexpression in the liver protects against diet-induced obesity and improved glucose homeostasis in mice. Moreover, our in vitro study demonstrated that NOTUM alleviated the inhibitory effects of WNT3A on brown adipocyte differentiation by attenuating Wnt/β-catenin signaling pathway and increasing the mRNA and protein levels of C/EBPα and PPARγ, which are important transcription factors for adipocyte differentiation. Furthermore, addition of NOTUM greatly increased mRNA levels of *Pgc1a* and *Prdm16*, important regulators of brown adipocyte differentiation, and the mRNA and protein levels of the thermogenic protein, UCP1*,* in WNT3A + NOTUM group compared to the WNT3A group. We postulate that NOTUM blocks the inhibitory effects of Wnts on brown adipocyte differentiation by removing an essential palmitoleate from Wnt proteins, leading to their inactivation^10^.

We observed that NOTUM overexpression was associated with decreased fasting insulin levels and improved glucose tolerance in obese mice. This is likely due to (1) increased WAT expression of adiponectin, a beneficial adipokine that sensitizes the body to insulin^24^, (2) decreased inflammation in WATs as evidenced by decreased expression of TNFα, a cytokine that promotes insulin resistance, and decreased CCL2 protein levels in eWAT, and (3) decreased fibrosis in WATs. Fibrosis in visceral fat is associated with insulin resistance in humans^80^. A recent publication identified General Transcription Factor II-I Repeat Domain-Containing Protein 1 (GTF2IRD1) as a cold-inducible transcription factor that represses adipose tissue fibrosis through a PRDM16-Euchromatic Histone Lysine Methyltransferase 1 (EHMT1) complex^81^. Repression of adipose tissue fibrosis by the complex improves systemic glucose homeostasis independent of UCP1-mediated thermogenesis and body weight in mice^81^, demonstrating a direct link between WAT fibrosis and insulin resistance. Consistent with reduced insulin resistance, AAV-Notum mice exhibited improved glucose tolerance, although we were unable to demonstrate statistically significant differences in insulin sensitivity by ITT, likely due to the relatively modest effect of Notum on this trait. A more sensitive hyperinsulinemic-euglycemic clamp study^82^ will be needed to further characterize this trait.

In the metabolic cage experiments performed at ambient temperature (23 °C), we did not observe significant differences in energy expenditure related traits between the AAV-GFP and AAV-Notum treated mice on the HF/HS diet for 6 weeks. However, our previous study demonstrated that acute overexpression of NOTUM in the mouse liver, through adenovirus mediated gene delivery, resulted in increased oxygen consumption and carbon dioxide production only when the mice were maintained at 5 °C, but not when the mice were kept at 23°C^9^. Therefore, the metabolic cage experiment we conducted at 23 °C in this study might not be sensitive enough to capture a subtle difference in whole body energy expenditure between the two groups of mice. The other possibility is that a short acclimation period (overnight) used in our metabolic cage protocol might not be long enough, leading to increased experimental variations and decreased sensitivity to detect differences between the groups.

We observed that NOTUM overexpression was associated with increased fasting plasma FFA levels, increased ATGL and HSL mRNA and protein levels in WAT, and increased β3 adrenergic receptor agonist stimulated lipolysis capacity. The increased *Atgl* and *Lipe* (gene encoding HSL) transcript levels observed in the iWAT and eWAT of AAV-Notum mice compared to AAV-GFP mice are likely due to increased PPARγ transactivation of these two PPARγ target genes^83,84^. Our in vitro data also demonstrated that NOTUM treatment led to increased *Atgl* and *Lipe* mRNA levels in the differentiated brown adipocytes. Another possible cause of increased *Atgl* mRNA levels in the AAV-Notum mice could be decreased Snail1 (encoded by *Snai1*) expression (Fig. 7a,b). Snail1, a Wnt target gene^85^, is a transcriptional repressor that is known to bind to the promoter of *Atgl* and repress its expression, leading to inhibition of lipolysis^86^. Thus, decreased *Snai1* expression in the AAV-Notum group due to inhibition of Wnt signaling might lead to increased *Atgl* expression. During fasting, WAT lipolysis is increased by a combination of decreased insulin level, which inhibits lipolysis, and increased release of adrenaline and noradrenaline from the adrenal gland^87^. Impaired lipolysis has been associated with obesity in humans^88,89^. Recent reports^90,91^ demonstrated that, under fasting condition, ATGL-mediated lipolysis from WAT but not from BAT is essential for supplying FFA for non-shivering thermogenesis in BAT. Thus, the increased WAT lipolysis and increased UCP1 protein level in BAT can act synergistically to enhance BAT thermogenic function in the AAV-Notum treated mice. In addition, since the mRNA levels of the fatty acid transporters, Cd36 and Slc27a1, were similar between WATs and iBATs of AAV-Notum and AAV-GFP mice, the observed differences in plasma FFA levels between the two groups of mice could not be attributed to differences in expression of FFA transporters in adipose tissues.

We observed decreased levels of plasma LDL- and HDL-cholesterol but no changes in triglycerides in the AAV-Notum mice compared to the AAV-GFP mice in the DIO experiment (Table 1). Although high plasma triglycerides and LDL-cholesterol, and low HDL-cholesterol levels are associated with type 2 diabetes and insulin resistance in humans^92,93^, the insulin resistance-associated dyslipidemia in animal models exhibits different features^94,95^. For example, male C57BL/6J mice fed a high fat diet for 16 weeks exhibited obesity, insulin resistance, increased plasma triglycerides, and increased HDL- and LDL-cholesterol levels as compared to the chow diet-fed mice^95^. Therefore, *increased*, but not decreased, HDL-cholesterol level is associated with insulin resistance in male C57BL/6J DIO model. Thus, the decreased HDL-cholesterol level in the AAV-Notum treated mice compared to AAV-GFP mice is probably not related to insulin resistance. Furthermore, there was no differences in hepatic expression of the HDL-cholesterol receptor, SRB1 (encoded by *Scarb1*), between the AAV-Notum and AAV-GFP groups, excluding SRB1 as a cause for decreased HDL-cholesterol in the former. We observed significantly decreased transcript levels of lipogenesis genes including *Fasn*, *Me1*, and *Scd1* in the livers of AAV-Notum mice compared to AAV-GFP mice. This is probably due to decreased circulating insulin levels in the former. Therefore, lower insulin levels in AAV-Notum mice lead to less activation of Srebp-1c, resulting in reduced expression of lipogenesis genes in the liver. The opposing effects of decreased hepatic lipogenesis and increased circulating FFA levels in the AAV-Notum mice compared to the AAV-GFP mice probably led to no differences in plasma or liver triglyceride levels between the two groups.

Our iWAT RNA-seq data revealed a very significant enrichment of the ECM functional cluster among the down-regulated genes associated with NOTUM overexpression. ECM is important for maintaining adipose tissue homeostasis. Excessive accumulation of the ECM in adipose tissues causes fibrosis, which is associated with insulin resistance and type 2 diabetes^81,96–101^. ECM components such as collagen I, III, V and VI have been shown to be upregulated in obese mice^102–104^. The transforming growth factor-β (TGF-β) signaling pathway is a key mediator of activating fibroblast. Canonical Wnt signaling activation is required for TGF-β-mediated fibrosis and TGF-β can also stimulate canonical Wnt signaling^105^. Canonical Wnt pathway signaling is known to contribute to fibrotic mechanisms in various tissues and in cultured cells^67,69^. Interestingly, Wnt signaling has been shown to stimulate fibrogenic responses while suppressing adipogenesis in cultured human preadipocytes^106^. Global suppression of Wnt signaling pathways through chemical inhibition of the Wnt-activating enzyme, porcupine, leads to decreased expression of profibrotic genes such as collagen genes in the heart under normal basal conditions and improvement of heart function and reduced scaring following myocardial infarction^107^. A recent report demonstrated that NOTUM inhibited Wnt family member 5a (*Wnt5a*)-induced expression of profibrotic genes such as α-SMA, TIMP-1, and COL1A1 in a stellate cell line^108^. Furthermore, the C-terminal portion of COL6A3 collagen protein, known as endotrophin, has been shown to trigger adipose tissue fibrosis, inflammation, and insulin resistance in mice^109^. Our RNA-seq data showed that *Col6a3* mRNA level was decreased in the iWAT of AAV-Notum treated mice (Fig. 7d), which might contribute to improved insulin resistance. Our in vitro data from brown pre-adipocytes further demonstrated that NOTUM blocked upregulation of TGF-β signaling by WNT3A, leading to significant decreases in mRNA levels of multiple collagen genes that are downstream targets of TGF-β signaling^70,71^. We postulate that NOTUM decreases adipose tissue fibrosis through inhibition of Wnt signaling in vivo as well.

In summary, our study demonstrates that NOTUM overexpression in the liver protects against diet-induced obesity and improves glucose homeostasis in mice, probably through enhanced thermogenic capacity and decreased fibrosis and inflammation in the adipose tissue. Our in vitro studies provide further evidence that NOTUM blocks Wnt signaling, leading to restored brown adipocyte differentiation and decreased expression of fibrosis related genes. Our results suggest that NOTUM is a potential therapeutic target for the treatment of obesity and diabetes.

### Limitations of study

This work did not include a saline-receiving group as another control in the animal studies, which would have provided information regarding whether overexpression of GFP in the liver of the AAV-GFP injected mice caused adverse effects, such as immunogenicity and cytotoxicity^110^, which might change interpretation of the data. We did not measure heat production or core body temperature following cold exposure in animal studies, nor did we study DIO under thermal neutral vs. cold temperature conditions, therefore, preventing us to demonstrate directly that NOTUM overexpression increases thermogenesis in BAT and WAT. Lastly, we did not measure triglyceride levels or lipolysis in the differentiated brown adipocytes, which would have further validated our in vitro model and in vivo observations.

## Material and methods

### AAV production

Recombinant adeno-associated virus (AAV) serotype 8 (AAV) vectors that overexpress mouse *Notum* or green fluorescence protein (GFP) under the control of a liver specific thyroxine binding globulin promoter were constructed using the same method as previously described^9^.

### Mice and diets

All mice included in this study were kept at 23 °C. For diet-induced obesity studies, eight-week-old male C57BL/6J mice purchased from the Jackson Laboratory (Bar Harbor, ME) were injected through the tail vein with 1 × 10^12^ genome copies of AAV-GFP or AAV-Notum (n = 12/group). Two weeks post injection, mice were switched to a high fat/high sucrose (HF/HS) diet (D12266B, Research Diets, New Brunswick, NJ) for 14 weeks, followed by 4-h fasting, before euthanization and tissue collection. Two cohorts of mice (n = 24 per cohort: 12 AAV-GFP and 12 AAV-Notum) were examined using the 14-week HF/HS diet feeding protocol with similar results. For the chow diet study, mice were infected with the same amounts of viruses as described above (n = 6/group), and fed a low fat chow diet for 4 weeks before euthanization and tissue collection as described above. The use of animals and all experimental procedures were reviewed and approved by the UCLA Chancellor’s Animal Research Committee and conducted in accordance with the animal care guideline set by UCLA. This study was carried out in compliance with the ARRIVE guidelines (https://arriveguidelines.org).

### Body composition by quantitative nuclear magnetic resonance

Mice were measured for total body fat mass and lean mass by nuclear magnetic resonance (NMR) using the Bruker Minispec with software from Echo Medical Systems (Houston, TX) as described^111^.

### Cumulative food intake, food consumption and fecal energy content

For cumulative food intake, mice that received the same AAV were housed 4 mice/cage, and the amounts of diet consumed each week were recorded. Broken up food crumbs that fell onto the cage bottoms were recovered, weighed, and subtracted from the food intake calculation to increase accuracy of the measurement. In a separate experiment, after 5-week diet feeding, before differences in body weight appeared, mice were individually housed for 48 h during which body weights and food weights were recorded at time 0, 24, and 48 h. Broken up food crumbs were recovered and subtracted from the food consumption calculation. Feces were collected and weighed at the end of 48 h. Energy content of food and feces was determined using a bomb calorimeter (Model 1261, Parr Instruments, Moline, IL) as previously described^112^. Samples were dried and the energy content of the dried samples were measured in duplicate and then averaged. Net energy intake of each mouse, expressed as kcal/day), was calculated as [daily food consumption (g/day)* energy content of diet (4.782 kcal/g)] − [fecal weight (g/day)* energy content of feces (kcal/g)].

### Metabolic cage study

Energy expenditure was assessed by indirect calorimetry using a Columbus Instruments Comprehensive Lab Animal Monitoring System as previously described^9^. Animals were placed individually in cages at 23 °C for 3 days with 12-h light/dark cycles with free access to food and water. All of the parameters, such as oxygen (VO_2_) and carbon dioxide (CO_2_), were measured in 20-min intervals after an overnight acclimation period. Data collected from the metabolic cage study after overnight acclimation were analyzed using the web-based CalR software version 1.2 (https://calrapp.org/)^19^. Body weight, lean mass, or fat mass of the mice were used as a covariate, separately, when performing ANCOVA.

### Blood parameters

Plasma lipids, lipoproteins, and glucose levels were measured by colorimetric assays as previously described^113^. Plasma insulin levels were measured by ELISA using kits from ALPCO (Salem, NH)^113^.

### Glucose tolerance and insulin tolerance tests

For glucose tolerance test, after 12 weeks feeding of the HF/HS diet, mice were fasted for 6 h before the intraperitoneal glucose tolerance test (IPGTT). For the test, 1.5 g glucose/kg body weight of glucose was injected intraperitoneally (i.p.) into each mouse. Blood glucose levels were determined at time 0, 15, 30, 60, 90, and 120 min using one drop of blood collected from the tip of the tail and an Alphatrak 2 glucometer^114^.

For insulin tolerance test (ITT), mice maintained on the HF/HS diet for 11 weeks were fasted for 4 h before receiving i.p. injection of recombinant human insulin (1U/kg body weight, Gibco, Carlsbad, CA). Blood glucose levels were determined at time 0, 15, 30, 60, and 90 min using an Alphatrak 2 glucometer^115^. Glucose values at time 0, 15, 30, 60, and 90 min were normalized to values at time 0 of each mouse for data analysis.

For IPGTT and ITT, area under the curve (AUC) for glucose or glucose (%) of each mouse, respectively, was calculated using Prism 8.0.2 (GraphPad Software Inc., La Jolla, CA).

### β3 adrenergic receptor agonist-stimulated lipolysis test

Mice maintained on the HF/HS diet for 10 weeks were injected i.p. with a β3 adrenergic receptor agonist, CL316,243 (0.3 mg/kg body weight, Adipogen, San Diego, CA)^116^ at 10 a.m. Blood were collected at time 0 and 30 min after injection. Plasma free fatty acid and glycerol levels were determined as previously described^113^.

### RNA purification, cDNA synthesis, and real-time RT-PCR

Total RNA was isolated from tissue samples using the miRNA isolation kit (Qiagen, Germantown, MD) according to protocol provided by the manufacturer. One μg of RNA samples were reversely transcribed using a high-capacity cDNA reverse transcription kit (Applied Biosystems, Foster City, CA) with random primers. Real-time qPCR was carried out using a KAPA SYBR Fast qPCR kit (Kapa Biosystems), as recommended by the manufacturer. Samples were run on a LightCycler 480 II (Roche) and analyzed using the Roche LightCycler 480 software version 1.5.0. The qPCR targets were normalized to the expression of housekeeping genes including *Hmbs*, *Nono*, and *Ywhaz*. Primer sequences for qPCR are listed in Supplemental Table 4.

### Immunoblotting

Immunoblotting was performed using 30 μg of proteins from tissue or cell lysate per sample as previously described^117^. Primary antibodies against CALNEXIN (Santa Cruz Biotechnology, 1:1000), active β-CATENIN (Cell Signaling, 1:1000), APOE (Biodesign International, 1:1000), ATGL (Cell Signaling, 1:1000), C/EBPα (Cell Signaling, 1:1000), GAPDH (Cell Signaling, 1:1000), HSL (Cell Signaling, 1:1000), PPARγ (Cell Signaling, 1:000), SMAD2 (Cell Signaling, 1:1000), phospho-SMAD2 (Cell Signaling, 1:1000), UCP1 (abcam, 1:1000), NOTUM (abcam, 1:1000), and β-ACTIN (Cell Signaling, 1:2000) were used in the experiments.

### Mitochondrial DNA content

Mitochondrial DNA content was measured as described previously^118,119^. Briefly, total (mitochondrial and nuclear) DNA from various adipose tissues was isolated by phenol/chloroform/isoamylalcohol extraction. Both mitochondrial and nuclear DNAs were amplified by quantitative PCR with 25 ng of total DNA using primers in the D-loop region and Tert gene (Supplemental Table 4), respectively. Mitochondrial DNA content, normalized to nuclear DNA, was calculated using the equation 2 × 2^−ΔCt^, ΔCt = (D-loop Ct –Tert Ct).

### Histology and adipocyte size and number measurements

Histologic sections (5 μm) from formalin fixed paraffin embedded mouse iBAT and iWAT were stained with hematoxylin and eosin (H & E) stain before examination using a light microscope. H & E stained histological sections of iWATs were used for determination of adipocyte size^120^ in a blinded fashion. Determination of adipocyte number was performed as described^121,122^.

#### CCL2 ELISA

One hundred μl of tissue lysate from epididymal WAT (eWAT) [in Pierce IP buffer (Thermo Fisher)] was used for determination of CCL2 content, in duplicate, using the mouse CCL2 DuoSet ELISA kit (R&D Systems) according to the manufacturer’s protocol. CCL2 concentration was normalized by protein concentration of the tissue lysate.

### Collagen assay

Hydroxyproline content of adipose tissue samples was measured using QuickZyme Total Collagen Assay kit (QuickZyme Biosciences, The Netherlands).

### RNA-seq

Total RNA from iWAT was isolated as described above. RNA libraries were prepared using the Illumina TruSeq kits (Illumina, San Diego, CA). Following barcoding, 12 samples per lane were sequenced on a HiSeq4000 using 50 bp single-end protocol. Reads were QC’d using FastQC in batch mode and mapped to the mouse genome (mm10) using STAR aligner version 2.3.1. Differential expression analysis was performed using DEseq2^123^ with significant genes called using adjusted p-value cutoffs of less than 0.05. Gene ontology analysis was performed using DAVID 6.8 (https://david.ncifcrf.gov/). The Benjamini–Hochberg method was used to obtain false discovery rates for enriched pathways.

### In vitro study

Confluent brown pre-adipocytes^9^ plated in 6-well plates were subjected to differentiation using a standard cocktail^9^ with additions of BSA (1 μg/ml, control), 10 ng/ml recombinant human WNT3A (R&D Systems), 300 ng/ml recombinant human NOTUM (R&D Systems), or 10 ng/ml WNT3A and 300 ng/ml NOTUM for 7 days before gene expression analysis and immunoblotting. Cell samples were also collected at baseline (Day 0) to serve as references. Media were changed every 2 days during the 7-day period. The WNT3A protein stock solution was dissolved in PBS containing 0.1% BSA at a concentration of 10 μg/ml as recommended by the manufacturer. Final BSA concentrations in all treatment groups are 1 μg/ml.

### Statistical analysis

Student’s t-test was used to compare the means of 2 groups. For experiments with a two-factorial design (data presented in Fig. 5), a two-way ANOVA was performed to establish that not all groups were equal. The Holm–Sidak post hoc analysis was then used for specific between-group comparisons after statistical significance was established by ANOVA. Statistical analyses were performed in GraphPad Prism version 8.0.2.

## Supplementary Information

Supplementary Information.

## Data Availability

All data are contained within the manuscript. The RNA-seq data presented in this publication have been deposited in NCBI's Gene Expression Omnibus^124^ and are accessible through GEO Series accession number GSE148894 (https://www.ncbi.nlm.nih.gov/geo/query/acc.cgi?acc=GSE148894).
