# Supplementary material for: NOTUM promotes thermogenic capacity and protects against diet-induced obesity in male mice
Source: Sci Rep. 2021 Aug 12;11:16409. doi: 10.1038/s41598-021-95720-1 (PMC8361163; doi:10.1038/s41598-021-95720-1)

SUPPLEMENTARY INFORMATION

Title: NOTUM promotes thermogenic capacity and protects against diet-induced obesity in male mice

Authors: Fangfei Guo[1], Marcus Seldin [2], Miklós Péterfy [3], Sarada Charugundla [1], Zhiqiang Zhou [1], Stephen D. Lee [4], Alice Mouton [5], Prashant Rajbhandari [6], Wenchao Zhang [1,7], Matteo Pellegrini [8], Peter Tontonoz [4], Aldons J. Lusis [1], and Diana M. Shih [1 *]

Supplemental Table 1. Expressed Wnt target genes in iWAT of chow fed AAV-Notum and AAV-GFP mice

Gene Name	log2 fold change	p value
Egfr	-0.87	4.63E-04
Jun	-0.91	3.74E-03
Efnb1	-0.55	5.97E-03
Bmp4	-0.55	8.41E-03
Vcan	-0.82	1.36E-02
Wisp2	-0.81	1.57E-02
Rarg	-0.34	3.06E-02
Snai1	-0.49	3.15E-02
Mmp3	-0.35	4.82E-02
Dll1	-0.29	5.16E-02
Tbx3	-0.46	5.45E-02
En1	-0.72	5.45E-02
Grem1	1.42	5.92E-02
Fzd5	-0.42	6.80E-02
Birc5	0.97	8.03E-02
Jag1	-0.38	9.17E-02
Enpp2	-0.21	9.78E-02
Edn1	-0.59	1.01E-01
Nrcam	-0.40	1.03E-01
Mmp2	-0.36	1.08E-01
Tnfsf9	0.60	1.20E-01
Tcf7	0.97	1.53E-01
Ctla4	1.10	1.56E-01
Fzd7	-0.32	1.56E-01
Mitf	-0.43	1.63E-01
Eda	-0.42	1.75E-01
Lbh	0.63	1.79E-01
Irx3	-0.23	1.82E-01
Vegfa	-0.31	1.83E-01
Myc	0.40	2.00E-01
Nos2	-0.30	2.02E-01
Mmp9	-0.45	2.14E-01
Tcf4	-0.17	2.18E-01
Tnfsf11	0.67	2.30E-01
Postn	0.38	2.33E-01

Supplemental Table 1-continued

Gene Name	log2 fold change	p value
Btrc	-0.11	2.68E-01
Tert	0.35	2.94E-01
Gja1	0.30	3.05E-01
Igf1	-0.21	3.29E-01
Ccnd1	0.24	4.34E-01
Plaur	0.29	4.81E-01
Axin2	-0.10	5.12E-01
Mycbp	-0.14	5.16E-01
Cacna1g	-0.18	5.85E-01
Ikbkg	-0.07	5.91E-01
Fst	0.12	6.36E-01
Msl1	0.04	6.95E-01
Vegfc	-0.07	7.09E-01
Wisp1	0.18	7.57E-01
Rhou	0.09	7.64E-01
Cldn1	-0.17	7.64E-01
L1cam	0.06	8.18E-01
Fn1	-0.04	9.01E-01
Sfrp2	-0.04	9.16E-01
Cdh1	-0.08	9.20E-01

Supplemental Table 2. DAVID pathway analysis-enriched cluster of upregulated genes in iWAT of AAV-Notum mice compared to AAV-GFP mice

Enrichment Cluster	Gene	log2 Fold Change	P value	P adjusted
**Cellular response to interferon-beta**	Gbp6	0.59	1.91E-04	1.84E-02
	Cdc34	0.36	3.93E-04	2.74E-02
	Igtp	0.61	1.17E-04	1.34E-02
	Stat1	0.53	1.26E-04	1.41E-02
	Ifit3	0.78	3.91E-04	2.74E-02
	Gbp3	0.52	7.92E-09	1.18E-05

Supplemental Table 3. DAVID pathway analysis-enriched clusters of down-regulated genes in iWAT of AAV-Notum mice compared to AAV-GFP mice

Enrichment Cluster	Gene	log2 Fold Change	P value	P adjusted
**Glycoprotein (120 genes)**	Abca8a	-0.85	2.17E-04	1.91E-02
	Abcb1a	-0.51	5.08E-04	3.07E-02
	Abcc3	-0.98	3.76E-05	6.44E-03
	Acer2	-0.74	1.72E-07	1.18E-04
	Acvr1	-0.87	8.77E-04	4.22E-02
	Acvrl1	-0.49	1.78E-04	1.80E-02
	Adamts1	-0.83	7.23E-04	3.79E-02
	Adamts2	-0.78	9.67E-04	4.47E-02
	Adamtsl2	-1.87	2.34E-06	9.07E-04
	Adgrg1	-0.72	1.53E-04	1.66E-02
	Adgrg2	-0.96	3.49E-05	6.10E-03
	App	-0.53	1.00E-03	4.59E-02
	Aqp1	-0.74	4.24E-05	6.88E-03
	Asgr2	-1.47	1.10E-03	4.86E-02
	Bcam	-0.56	2.69E-04	2.19E-02
	Bmper	-0.72	8.84E-04	4.22E-02
	Btc	-1.86	6.98E-08	5.92E-05
	C1qtnf1	-0.55	8.59E-04	4.20E-02
	Ccdc80	-0.98	4.12E-04	2.78E-02
	Ccl11	-1.01	4.29E-05	6.89E-03
	Ccl2	-1.05	7.90E-05	1.07E-02
	Cd163	-0.92	8.61E-08	6.98E-05
	Cmklr1	-1.00	9.99E-07	4.82E-04
	Cnnm3	-0.43	7.27E-04	3.80E-02
	Col18a1	-0.87	4.04E-04	2.75E-02
	Col1a1	-0.52	1.12E-03	4.89E-02
	Col1a2	-0.57	2.73E-04	2.21E-02
	Col4a1	-0.70	3.98E-04	2.75E-02
	Col4a2	-0.69	1.01E-03	4.61E-02
	Col6a1	-0.72	1.24E-04	1.40E-02
	Col6a2	-0.68	4.23E-04	2.80E-02
	Creb3l1	-0.72	7.54E-04	3.85E-02
	Creb3l3	-1.94	9.96E-04	4.58E-02
	Crim1	-0.71	8.38E-06	2.26E-03
	Ddr2	-0.48	4.70E-04	2.93E-02
	Duoxa1	-1.32	9.51E-05	1.19E-02

Supplemental Table 3-continued

Enrichment Cluster	Gene	log2 Fold Change	P value	P adjusted
**Glycoprotein-continued**	Egfr	-0.80	9.56E-08	7.09E-05
	Emp1	-1.93	3.28E-07	1.95E-04
	Erbb2	-0.65	7.39E-04	3.82E-02
	Fam20a	-0.80	1.71E-04	1.77E-02
	Fbln2	-0.68	8.30E-04	4.15E-02
	Fbln5	-0.98	5.47E-06	1.70E-03
	Folh1	-2.71	6.34E-06	1.87E-03
	Fzd1	-0.63	3.03E-06	1.08E-03
	Gcnt2	-0.75	2.24E-05	4.53E-03
	Glt8d2	-0.97	1.05E-03	4.70E-02
	Gpr156	-1.53	1.18E-09	2.79E-06
	Gpr161	-0.60	4.89E-04	3.01E-02
	Gpr17	-1.04	1.64E-06	7.48E-04
	Gpr4	-0.51	6.50E-04	3.56E-02
	H6pd	-0.80	3.28E-04	2.43E-02
	Heg1	-0.47	2.11E-04	1.90E-02
	Hmcn2	-0.74	5.26E-04	3.14E-02
	Hspg2	-0.79	8.81E-05	1.13E-02
	Il34	-0.80	1.13E-03	4.93E-02
	Itga5	-0.49	4.21E-05	6.88E-03
	Itih5	-1.00	1.11E-04	1.28E-02
	Jag2	-0.57	6.44E-04	3.56E-02
	Jup	-0.76	1.65E-04	1.75E-02
	Kcnq1	-0.93	5.93E-04	3.42E-02
	Kirrel	-0.87	1.74E-04	1.78E-02
	Kitl	-0.73	1.12E-03	4.89E-02
	Lama5	-0.54	2.67E-04	2.19E-02
	Lamb2	-0.87	4.17E-05	6.88E-03
	Lamc1	-0.60	8.65E-04	4.21E-02
	Lbp	-1.81	2.21E-07	1.40E-04
	Lox	-1.13	2.05E-07	1.35E-04
	Lrp6	-0.54	2.57E-04	2.13E-02
	Mest	-2.88	1.50E-10	5.36E-07
	Nid1	-0.78	8.93E-04	4.24E-02
	Notch3	-0.31	5.07E-04	3.07E-02
	Notch4	-0.59	2.20E-04	1.91E-02

Supplemental Table 3-continued

Enrichment Cluster	Gene	log2 Fold Change	P value	P adjusted
**Glycoprotein-continued**	Npr3	-1.75	2.05E-04	1.90E-02
	Olfml3	-0.75	7.91E-05	1.07E-02
	P2ry2	-1.03	3.19E-06	1.09E-03
	P4ha1	-0.55	4.04E-04	2.75E-02
	P4ha2	-0.76	4.39E-04	2.85E-02
	Pam	-0.40	1.15E-03	4.96E-02
	Pcdh19	-0.53	2.32E-04	1.99E-02
	Pcsk5	-1.00	3.99E-04	2.75E-02
	Plxdc2	-0.76	6.86E-05	9.71E-03
	Pon1	-1.12	5.41E-04	3.20E-02
	Prelp	-1.46	6.09E-06	1.84E-03
	Prss23	-0.58	1.11E-03	4.88E-02
	Ptgfr	-2.26	5.53E-08	5.03E-05
	Ptgfrn	-0.37	6.28E-04	3.51E-02
	Ptprb	-0.68	8.94E-04	4.24E-02
	Ptprf	-0.46	4.23E-04	2.80E-02
	Ret	-1.07	1.16E-04	1.33E-02
	Robo4	-0.64	6.77E-04	3.67E-02
	Sema3f	-0.77	6.00E-07	3.24E-04
	Sema5a	-0.73	1.02E-04	1.22E-02
	Serpine1	-2.36	1.00E-14	5.97E-11
	Serpinf1	-0.86	4.36E-04	2.85E-02
	Serping1	-0.47	2.54E-06	9.63E-04
	Serpinh1	-0.73	2.09E-04	1.90E-02
	Sez6l	-3.24	8.26E-05	1.09E-02
	Slc1a4	-0.87	2.67E-05	4.96E-03
	Slc43a3	-0.67	1.99E-06	8.36E-04
	Slc4a11	-0.58	8.02E-04	4.03E-02
	Slc5a3	-0.96	1.62E-04	1.74E-02
	Slc5a7	-4.39	9.96E-06	2.54E-03
	Slc6a13	-1.26	2.14E-04	1.90E-02
	Slc8b1	-0.56	3.91E-04	2.74E-02
	Slit3	-0.91	2.07E-04	1.90E-02
	Smoc2	-1.11	2.15E-04	1.91E-02
	Sptbn1	-0.64	3.99E-04	2.75E-02
	Srpx2	-1.06	6.15E-04	3.48E-02
	Tanc2	-0.56	9.57E-05	1.19E-02

Supplemental Table 3-continued

**Enrichment Cluster**	**Gene**	**log2 Fold Change**	**P value**	**P adjusted**
**Glycoprotein-continued**	Tfpi2	-0.88	4.51E-04	2.87E-02
	Tgfb3	-0.63	1.74E-04	1.78E-02
	Thbd	-1.20	9.89E-06	2.54E-03
	Thbs1	-1.51	3.11E-04	2.38E-02
	Thbs2	-1.16	2.65E-15	2.36E-11
	Tinagl1	-0.93	1.18E-05	2.93E-03
	Tspan4	-0.65	3.04E-04	2.35E-02
	Ugt1a6a	-0.98	2.23E-04	1.92E-02
	Vcan	-0.78	7.03E-04	3.73E-02
	Vnn1	-1.23	3.06E-04	2.35E-02
	Vsig2	-0.81	4.19E-04	2.80E-02

**Enrichment Cluster**	**Gene**	**log2 Fold Change**	P value	**P adjusted**
**Extracellular matrix (34 genes)**	Adamts1	-0.83	7.23E-04	3.79E-02
	Adamts2	-0.78	9.67E-04	4.47E-02
	Adamtsl2	-1.87	2.34E-06	9.07E-04
	Ccdc80	-0.98	4.12E-04	2.78E-02
	Col18a1	-0.87	4.04E-04	2.75E-02
	Col1a1	-0.52	1.12E-03	4.89E-02
	Col1a2	-0.57	2.73E-04	2.21E-02
	Col4a1	-0.70	3.98E-04	2.75E-02
	Col4a2	-0.69	1.01E-03	4.61E-02
	Col5a1	-0.67	4.58E-04	2.90E-02
	Col6a1	-0.72	1.24E-04	1.40E-02
	Col6a2	-0.68	4.23E-04	2.80E-02
	Col6a3	-0.81	6.95E-06	1.94E-03
	Eln	-0.58	1.36E-06	6.37E-04
	Fbln2	-0.68	8.30E-04	4.15E-02
	Fbln5	-0.98	5.47E-06	1.70E-03
	Fgf1	-1.17	4.48E-04	2.87E-02
	Hapln3	-1.22	1.48E-04	1.62E-02
	Hmcn2	-0.74	5.26E-04	3.14E-02
	Hspg2	-0.79	8.81E-05	1.13E-02
	Lama5	-0.54	2.67E-04	2.19E-02
	Lamb2	-0.87	4.17E-05	6.88E-03
	Lamc1	-0.60	8.65E-04	4.21E-02

Enrichment Cluster	Gene	log2 Fold Change	P value	P adjusted
Extracellular matrix (34 genes)	Adamts1	-0.83	7.23E-04	3.79E-02
	Adamts2	-0.78	9.67E-04	4.47E-02
	Adamtsl2	-1.87	2.34E-06	9.07E-04
	Ccdc80	-0.98	4.12E-04	2.78E-02
	Col18a1	-0.87	4.04E-04	2.75E-02
	Col1a1	-0.52	1.12E-03	4.89E-02
	Col1a2	-0.57	2.73E-04	2.21E-02
	Col4a1	-0.70	3.98E-04	2.75E-02
	Col4a2	-0.69	1.01E-03	4.61E-02
	Col5a1	-0.67	4.58E-04	2.90E-02
	Col6a1	-0.72	1.24E-04	1.40E-02
	Col6a2	-0.68	4.23E-04	2.80E-02
	Col6a3	-0.81	6.95E-06	1.94E-03
	Eln	-0.58	1.36E-06	6.37E-04
	Fbln2	-0.68	8.30E-04	4.15E-02
	Fbln5	-0.98	5.47E-06	1.70E-03
	Fgf1	-1.17	4.48E-04	2.87E-02
	Hapln3	-1.22	1.48E-04	1.62E-02
	Hmcn2	-0.74	5.26E-04	3.14E-02
	Hspg2	-0.79	8.81E-05	1.13E-02

Supplemental Table 3-continued

Enrichment Cluster	Gene	log2 Fold Change	P value	P adjusted
**Extracellular matrix-continued**	Lox	-1.13	2.05E-07	1.35E-04
	Nid1	-0.78	8.93E-04	4.24E-02
	Prelp	-1.46	6.09E-06	1.84E-03
	Slit3	-0.91	2.07E-04	1.90E-02
	Smoc2	-1.11	2.15E-04	1.91E-02
	Tgfb3	-0.63	1.74E-04	1.78E-02
	Thbs2	-1.16	2.65E-15	2.36E-11
	Timp3	-0.85	3.07E-06	1.08E-03
	Timp4	-1.73	2.21E-05	4.52E-03
	Tnxb	-0.72	6.13E-04	3.48E-02
	Vcan	-0.78	7.03E-04	3.73E-02
**Enrichment Cluster**	**Gene**	**log2 Fold Change**	**P value**	**P adjusted**
**ECM-receptor interaction (16 genes)**	Col1a1	-0.52	1.12E-03	4.89E-02
	Col1a2	-0.57	2.73E-04	2.21E-02
	Col4a1	-0.70	3.98E-04	2.75E-02
	Col4a2	-0.69	1.01E-03	4.61E-02
	Col5a1	-0.67	4.58E-04	2.90E-02
	Col6a1	-0.72	1.24E-04	1.40E-02
	Col6a2	-0.68	4.23E-04	2.80E-02
	Col6a3	-0.81	6.95E-06	1.94E-03
	Hspg2	-0.79	8.81E-05	1.13E-02
	Itga5	-0.49	4.21E-05	6.88E-03
	Lama5	-0.54	2.67E-04	2.19E-02
	Lamb2	-0.87	4.17E-05	6.88E-03
	Lamc1	-0.60	8.65E-04	4.21E-02
	Thbs1	-1.51	3.11E-04	2.38E-02
	Thbs2	-1.16	2.65E-15	2.36E-11
	Tnxb	-0.72	6.13E-04	3.48E-02

Enrichment Cluster	Gene	log2 Fold Change	P value	P adjusted
Extracellular matrix-continued	Lox	-1.13	2.05E-07	1.35E-04
	Nid1	-0.78	8.93E-04	4.24E-02
	Prelp	-1.46	6.09E-06	1.84E-03

	Slit3	-0.91	2.07E-04	1.90E-02
	Smoc2	-1.11	2.15E-04	1.91E-02
	Tgfb3	-0.63	1.74E-04	1.78E-02
	Thbs2	-1.16	2.65E-15	2.36E-11
	Timp3	-0.85	3.07E-06	1.08E-03
	Timp4	-1.73	2.21E-05	4.52E-03
	Tnxb	-0.72	6.13E-04	3.48E-02
	Vcan	-0.78	7.03E-04	3.73E-02
**Enrichment Cluster**	**Gene**	**log2 Fold Change**	**P value**	**P adjusted**
ECM-receptor interaction (16 genes)	Col1a1	-0.52	1.12E-03	4.89E-02
	Col1a2	-0.57	2.73E-04	2.21E-02
	Col4a1	-0.70	3.98E-04	2.75E-02
	Col4a2	-0.69	1.01E-03	4.61E-02
	Col5a1	-0.67	4.58E-04	2.90E-02
	Col6a1	-0.72	1.24E-04	1.40E-02
	Col6a2	-0.68	4.23E-04	2.80E-02
	Col6a3	-0.81	6.95E-06	1.94E-03
	Hspg2	-0.79	8.81E-05	1.13E-02
	Itga5	-0.49	4.21E-05	6.88E-03
	Lama5	-0.54	2.67E-04	2.19E-02
	Lamb2	-0.87	4.17E-05	6.88E-03
	Lamc1	-0.60	8.65E-04	4.21E-02
	Thbs1	-1.51	3.11E-04	2.38E-02

	Thbs2	-1.16	2.65E-15	2.36E-11

Supplemental Table 3-continued

Enrichment Cluster	Gene	log2 Fold Change	P value	P adjusted
**Basement membrane (16 genes)**	Adamts1	-0.83	7.23E-04	3.79E-02
	Ccdc80	-0.98	4.12E-04	2.78E-02
	Col18a1	-0.87	4.04E-04	2.75E-02
	Col4a1	-0.70	3.98E-04	2.75E-02
	Col4a2	-0.69	1.01E-03	4.61E-02
	Col5a1	-0.67	4.58E-04	2.90E-02
	Hmcn2	-0.74	5.26E-04	3.14E-02
	Hspg2	-0.79	8.81E-05	1.13E-02
	Lama5	-0.54	2.67E-04	2.19E-02
	Lamb2	-0.87	4.17E-05	6.88E-03
	Lamc1	-0.60	8.65E-04	4.21E-02
	Nid1	-0.78	8.93E-04	4.24E-02
	Serpinf1	-0.86	4.36E-04	2.85E-02
	Smoc2	-1.11	2.15E-04	1.91E-02
	Thbs2	-1.16	2.65E-15	2.36E-11
	Timp3	-0.85	3.07E-06	1.08E-03
**Enrichment Cluster**	**Gene**	**log2 Fold Change**	**P value**	**P adjusted**
**Epidermal growth factor-like domain (22 genes)**	Adam3	-1.69	1.87E-04	1.84E-02
	Btc	-1.86	6.98E-08	5.92E-05
	Fbln2	-0.68	8.30E-04	4.15E-02
	Fbln5	-0.98	5.47E-06	1.70E-03
	Heg1	-0.47	2.11E-04	1.90E-02
	Hmcn2	-0.74	5.26E-04	3.14E-02
	Hspg2	-0.79	8.81E-05	1.13E-02
	Jag2	-0.57	6.44E-04	3.56E-02
	Lama5	-0.54	2.67E-04	2.19E-02
	Lamb2	-0.87	4.17E-05	6.88E-03
	Lamc1	-0.60	8.65E-04	4.21E-02
	Lrp6	-0.54	2.57E-04	2.13E-02
	Nid1	-0.78	8.93E-04	4.24E-02
	Notch3	-0.31	5.07E-04	3.07E-02
	Notch4	-0.59	2.20E-04	1.91E-02
	Pcsk5	-1.00	3.99E-04	2.75E-02
	Slit3	-0.91	2.07E-04	1.90E-02
	Thbd	-1.20	9.89E-06	2.54E-03

Enrichment Cluster	Gene	log2 Fold Change	P value	P adjusted
Basement membrane (16 genes)	Adamts1	-0.83	7.23E-04	3.79E-02
	Ccdc80	-0.98	4.12E-04	2.78E-02
	Col18a1	-0.87	4.04E-04	2.75E-02
	Col4a1	-0.70	3.98E-04	2.75E-02
	Col4a2	-0.69	1.01E-03	4.61E-02
	Col5a1	-0.67	4.58E-04	2.90E-02
	Hmcn2	-0.74	5.26E-04	3.14E-02
	Hspg2	-0.79	8.81E-05	1.13E-02
	Lama5	-0.54	2.67E-04	2.19E-02
	Lamb2	-0.87	4.17E-05	6.88E-03
	Lamc1	-0.60	8.65E-04	4.21E-02
	Nid1	-0.78	8.93E-04	4.24E-02
	Serpinf1	-0.86	4.36E-04	2.85E-02
	Smoc2	-1.11	2.15E-04	1.91E-02
	Thbs2	-1.16	2.65E-15	2.36E-11
	Timp3	-0.85	3.07E-06	1.08E-03
**Enrichment Cluster**	**Gene**	**log2 Fold Change**	**P value**	**P adjusted**
Epidermal growth factor-like domain (22 genes)	Adam3	-1.69	1.87E-04	1.84E-02
	Btc	-1.86	6.98E-08	5.92E-05
	Fbln2	-0.68	8.30E-04	4.15E-02
	Fbln5	-0.98	5.47E-06	1.70E-03
	Heg1	-0.47	2.11E-04	1.90E-02

	Hmcn2	-0.74	5.26E-04	3.14E-02
	Hspg2	-0.79	8.81E-05	1.13E-02
	Jag2	-0.57	6.44E-04	3.56E-02
	Lama5	-0.54	2.67E-04	2.19E-02
	Lamb2	-0.87	4.17E-05	6.88E-03
	Lamc1	-0.60	8.65E-04	4.21E-02
	Lrp6	-0.54	2.57E-04	2.13E-02
	Nid1	-0.78	8.93E-04	4.24E-02
	Notch3	-0.31	5.07E-04	3.07E-02
	Notch4	-0.59	2.20E-04	1.91E-02
	Pcsk5	-1.00	3.99E-04	2.75E-02
	Slit3	-0.91	2.07E-04	1.90E-02
	Thbd	-1.20	9.89E-06	2.54E-03
**Enrichment Cluster**	**Gene**	**log2 Fold Change**	**P value**	**P adjusted**
Epidermal growth factor-like domain-continued	Thbs1	-1.51	3.11E-04	2.38E-02
	Thbs2	-1.16	2.65E-15	2.36E-11

Supplemental Table 3-continued

Enrichment Cluster	Gene	log2 Fold Change	P value	P adjusted
**Epidermal growth factor-like domain-continued**	Thbs1	-1.51	3.11E-04	2.38E-02
	Thbs2	-1.16	2.65E-15	2.36E-11
	Tnxb	-0.72	6.13E-04	3.48E-02
	Vcan	-0.78	7.03E-04	3.73E-02
**Enrichment Cluster**	**Gene**	**log2 Fold Change**	**P value**	**P adjusted**
**PI3K-Akt signaling pathway (20 genes)**	Col1a1	-0.52	1.12E-03	4.89E-02
	Col1a2	-0.57	2.73E-04	2.21E-02

	Col4a1	-0.70	3.98E-04	2.75E-02
	Col4a2	-0.69	1.01E-03	4.61E-02
	Col5a1	-0.67	4.58E-04	2.90E-02
	Col6a1	-0.72	1.24E-04	1.40E-02
	Col6a2	-0.68	4.23E-04	2.80E-02
	Col6a3	-0.81	6.95E-06	1.94E-03
	Creb3l1	-0.72	7.54E-04	3.85E-02
	Creb3l3	-1.94	9.96E-04	4.58E-02
	Egfr	-0.80	9.56E-08	7.09E-05
	Fgf1	-1.17	4.48E-04	2.87E-02
	Itga5	-0.49	4.21E-05	6.88E-03
	Kitl	-0.73	1.12E-03	4.89E-02
	Lama5	-0.54	2.67E-04	2.19E-02
	Lamb2	-0.87	4.17E-05	6.88E-03
	Lamc1	-0.60	8.65E-04	4.21E-02
	Thbs1	-1.51	3.11E-04	2.38E-02
	Thbs2	-1.16	2.65E-15	2.36E-11
	Tnxb	-0.72	6.13E-04	3.48E-02

Supplemental Table 3-continued

Enrichment Cluster	Gene	log2 Fold Change	P value	P adjusted
**Developmental protein (32 genes)**	Adgrg1	-0.72	1.53E-04	1.66E-02
	Ang	-1.02	7.08E-06	1.94E-03
	Arl13b	-0.31	7.32E-04	3.81E-02
	Bicc1	-0.53	1.04E-04	1.23E-02
	Col18a1	-0.87	4.04E-04	2.75E-02
	Creb3l1	-0.72	7.54E-04	3.85E-02
	Dhh	-1.10	2.36E-05	4.59E-03
	Egfr	-0.80	9.56E-08	7.09E-05
	Enc1	-0.59	1.80E-04	1.80E-02
	Fgf1	-1.17	4.48E-04	2.87E-02
	Fzd1	-0.63	3.03E-06	1.08E-03
	Gpr161	-0.60	4.89E-04	3.01E-02
	Heg1	-0.47	2.11E-04	1.90E-02
	Hic1	-0.65	5.86E-04	3.40E-02
	Ifrd1	-0.56	8.83E-04	4.22E-02
	Jag2	-0.57	6.44E-04	3.56E-02
	Lhx6	-0.71	8.55E-04	4.20E-02
	Lrp6	-0.54	2.57E-04	2.13E-02
	Mturn	-1.10	2.16E-04	1.91E-02
	Nav1	-0.49	3.28E-05	5.79E-03
	Nkd1	-1.06	1.19E-04	1.35E-02
	Notch3	-0.31	5.07E-04	3.07E-02
	Notch4	-0.59	2.20E-04	1.91E-02
	Olfml3	-0.75	7.91E-05	1.07E-02
	Robo4	-0.64	6.77E-04	3.67E-02
	Sema5a	-0.73	1.02E-04	1.22E-02
	Sfrp5	-2.71	5.54E-08	5.03E-05
	Shroom3	-0.45	1.98E-04	1.87E-02
	Shroom4	-0.62	1.06E-03	4.75E-02
	Slit3	-0.91	2.07E-04	1.90E-02
	Spry1	-0.69	4.71E-05	7.28E-03
	Spry4	-1.36	3.97E-09	7.08E-06

Supplemental Table 4. PCR primers used in this study

Primer Name	Sequence		Primer Name	Sequence
Adipoq-F	GATGCAGGTCTTCTTGGTCCT		Mmp3-R	AGTCCTGAGAGATTTGCGCC
Adipoq-R	AGAGTCCCGGAATGTTGCAG		Nono-F	TGCTCCTGTGCCACCTGGTACTC
Bmp4-F	GAAGCTAGGTGAGTTCGGCA		Nono-R	CCGGAGCTGGACGGTTGAATGC
Bmp4-R	ATGGCACTACGGAATGGCTC		P2rx5-F	CTGCAGCTCACCATCCTGT
Ccl2-F	CACTCACCTGCTGCTACTCA		P2rx5-R	CACTCTGCAGGGAAGTGTCA
Ccl2-R	GCTTGGTGACAAAAACTACAGC		Pgc1a-F	TGAAAAAGCTTGACTGGCGTC
Cd36-F	TGGCACAGACGCAGCCTCCT		Pgc1a-R	CCGATTGGTCGCTACACCAC
Cd36-R	TGGAGGGGTGATGCAAAGGCA		Pparg-F	AAGATTTGAAAGAAGCGGTGAAC
Cd68-F	GACCTACATCAGAGCCCGAGT		Pparg-R	CTGTGTCAACCATGGTAATTTCAGT
Cd68-R	CGCCATGAATGTCCACTG		Prdm16-F	TGCGAAGGTGTCCAAACTGA
Cidea-F	AACCATGACCGAAGTAGCCG		Prdm16-R	CCTTGGGAGTGAAGTCCTCG
Cidea-R	CCAGGCCAGTTGTGATGACT		Rarg-F	AATATTTTCCGTGTCCCCGCC
Col1a1-F	GAGAGGTGAACAAGGTCCCG		Rarg-R	CCTGGTGGAAACTGCTGGAGG
Col1a1-R	AAACCTCTCTCGCCTCTTGC		Rb1-F	TGCATGGCTTTCAGATTCACC
Col3a1-F	GAGGAATGGGTGGCTATCCG		Rb1-R	GCTGAGAGGACAAGCAGGTT
Col3a1-R	TCGTCCAGGTCTTCCTGACT		Slc27a1-F	ACAAGCTGGATCAGGCAAGC
Col5a1-F	CTTCGCCGCTACTCCTGTTC		Slc27a1-R	CAGCTCTAGCCGAACACGAA
Col5a1-R	CCCTGAGGGCAAATTGTGAAAA		Snai1-F	CACACGCTGCCTTGTGTCT
Cox5b-F	GCGTTGTTAGACTCCCACCAA		Snai1-R	GGTCAGCAAAAGCACGGTT
Cox5b-R	AGCATATTGTATGGGTCCAGTCC		Tert-F	CTAGCTCATGTGTCAAGACCCTCTT
Cycs-F	ACCAGCCCGGAACGAATTAAA		Tert-R	GCCAGCACGTTTCTCTCGTT
Cycs-R	CCGAACAGACCGTGGAGATT		Tgfb1-F	AGCTGCGCTTGCAGAGATTA
Dio2-F	CAGTGTGGTGCACGTCTCCAA		Tgfb1-R	AGCCCTGTATTCCGTCTCCT
Dio2-R	TGAACCAAAGTTGACCACCAG		Tgfb3-F	ATGACCCACGTCCCCTATCA
D-loop-F	AATCTACCATCCTCCGTGAAACC		Tgfb3-R	CAGACGGCCAGTTCATTGTG
D-loop-R	TCAGTTTAGCTACCCCCAAGTTTAA		Tmem26-F	ACCCTGTCATCCCACAGAG
Efnb1-F	CTGGTCACTTGCAACAAGCC		Tmem26-R	TGTTTGGTGGAGTCCTAAGGTC
Efnb1-R	CACAGCATTTGGATCTTGCCC		Tnfa-F	CTGTAGCCCACGTCGTAGC
Egfr-F	GTCAGAGATGCGACCCTCAG		Tnfa-R	TTGAGATCCATGCCGTTG
Egfr-R	GTGCCTTGGCAGACTTTCTTT		Ucp1-F	CGATGTCCATGTACACCAAGGA
Esrra-F	GAGACTGAGACTGAACCCCC		Ucp1-R	TCGCAGAAAAGAAGCCACAA
Esrra-R	TGTACTCGATGCTCCCCTGG		Vcan-F	CAAGGATCTGCCGCTACCAG
Fabp4-F	TGAAATCACCGCAGACGACA		Vcan-R	GCTGGTTTCCATTTTGGCTTGA
Fabp4-R	ACACATTCCACCACCAGCTT		Wisp1-F	TGTGGGCTGTGTCCTGGAT
Hmbs-F	GATGGGCAACTGTACCTGACTG		Wisp1-R	CCTGACGCTGCAAAGGC
Hmbs-R	CTGGGCTCCTCTTGGAATG		Wisp2-F	CAGCCCAAGGACACCAACTT
Jun-F	GGGAGCATTTGGAGAGTCCC		Wisp2-R	GGATACTCGGGTGGCTATGC
Jun-R	TTTGCAAAAGTTCGCTCCCG		Ywhaz-F	AAAAACAGCTTTCGATGAAGCC
Lgr4-F	TGCTGCGGACTCTGGACTTA		Ywhaz-R	GCCGGTTAATTTTCCCCTCC
Lgr4-R	TCGCAAAAGCTCCACTGTGA		Znrf3-F	CAAGGCCAAGAGAGCAGTTC
Mmp3-F	GTCCCTCTATGGAACTCCCAC		Znrf3-R	GCACCCTTCACATACACCAC

Fig. 1a. **Uncropped images**. Blot was cut into 2 parts before antibody incubation.

NOTUM

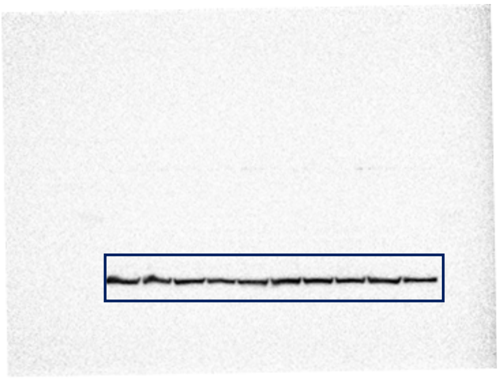

Fig. 1a, GAPDH, blot

GAPDH

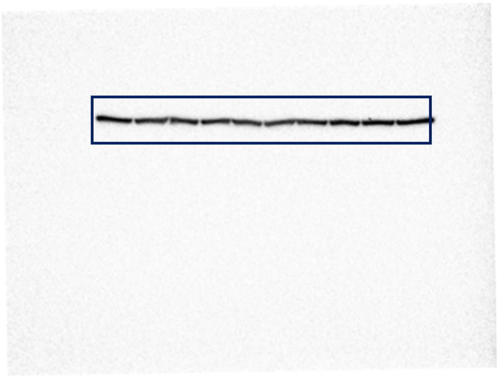

Fig. 1b. Uncropped images. Blot was cut into 2 parts before antibody incubation.

NOTUM

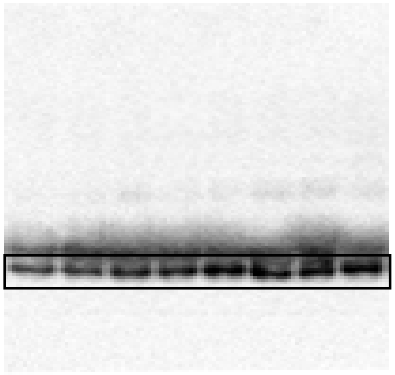

APOE

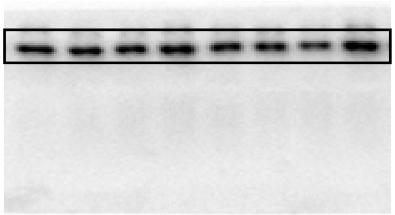

Fig. 3c. Uncropped images. Blot was cut into 3 parts before antibody incubation.

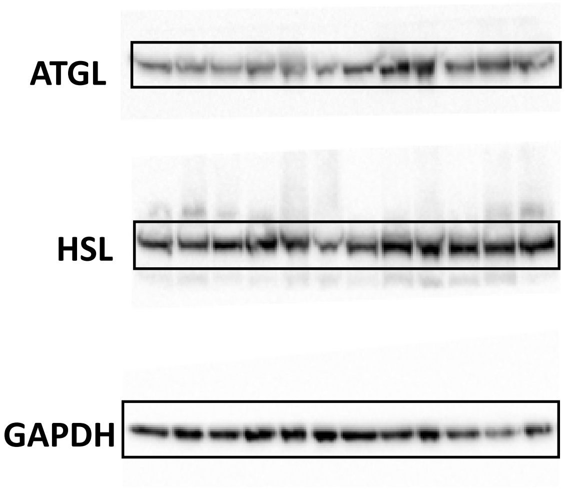

Fig. 4b. Uncropped images. Blot was cut into 2 parts before antibody incubation.

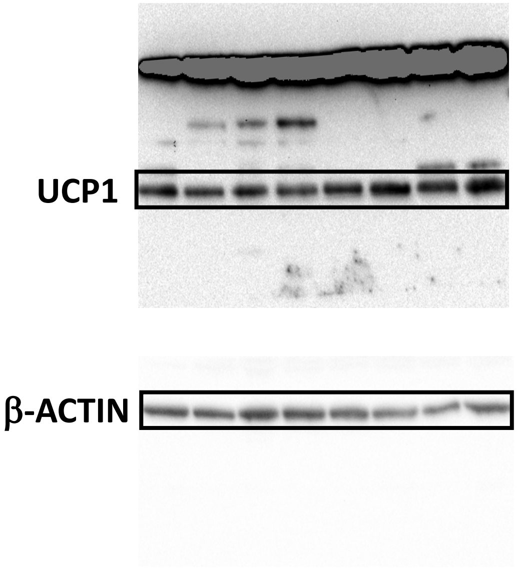

Fig. 4g. Uncropped images. Blot was cut into 2 parts before antibody incubation.

UCP1

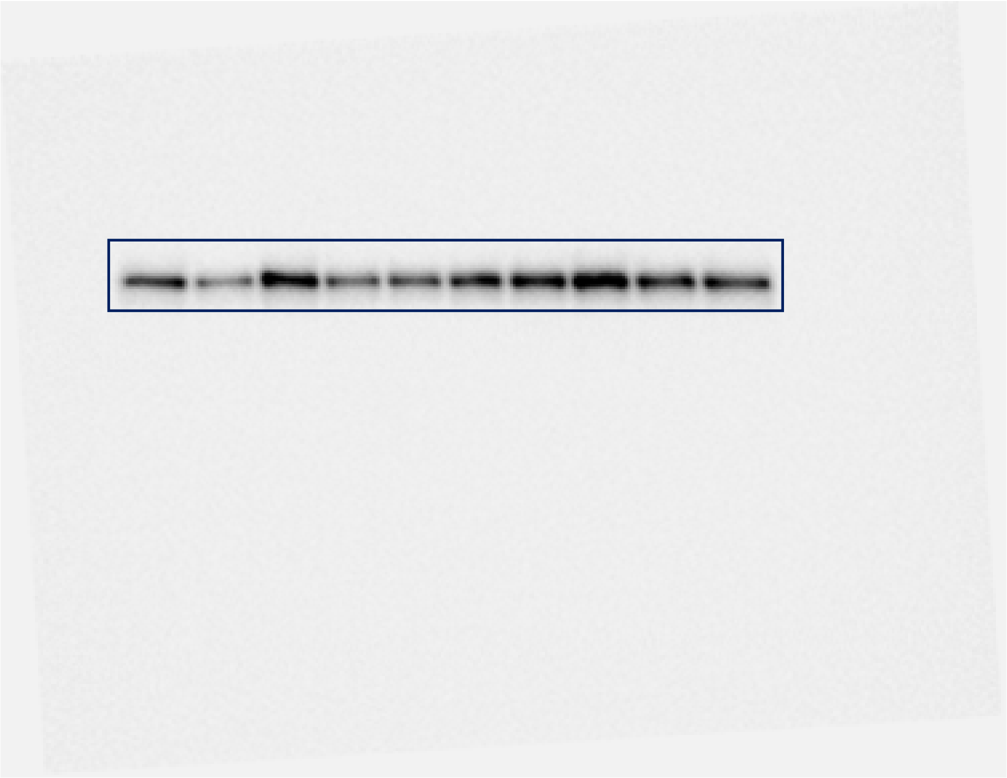

CALNEXIN

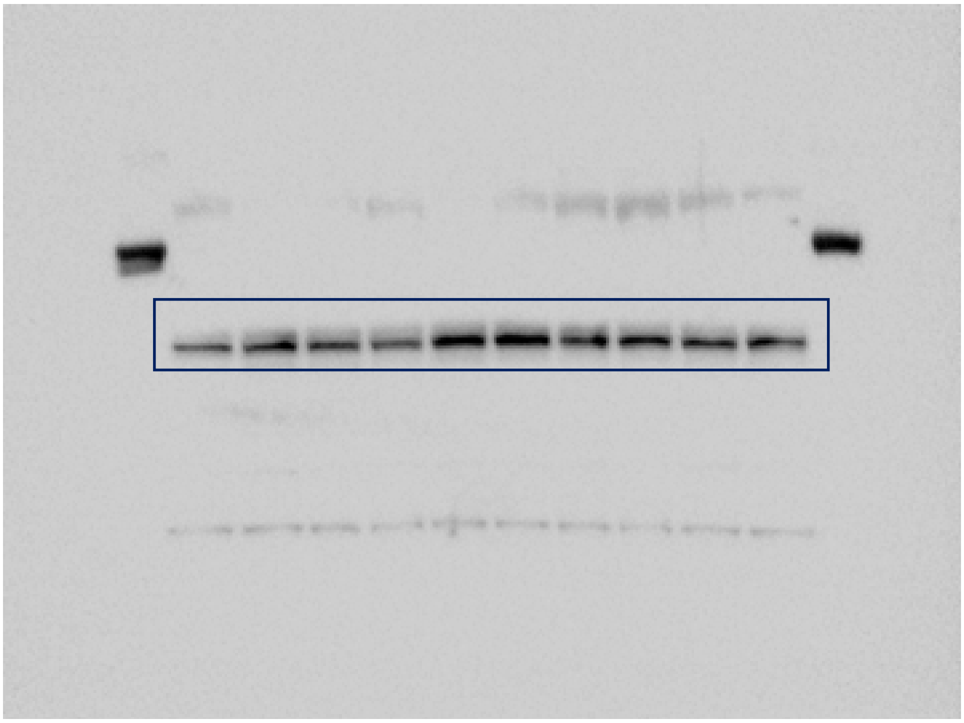

Fig. 8b. Uncropped images, top 4 panels. The images were obtained from the same blot: C/EBPa, PPARg, and b-ACTIN images were obtained from the top part of the same blot after stripping and re-probing, whereas image of UCP1 was obtained from the bottom part of the same blot.

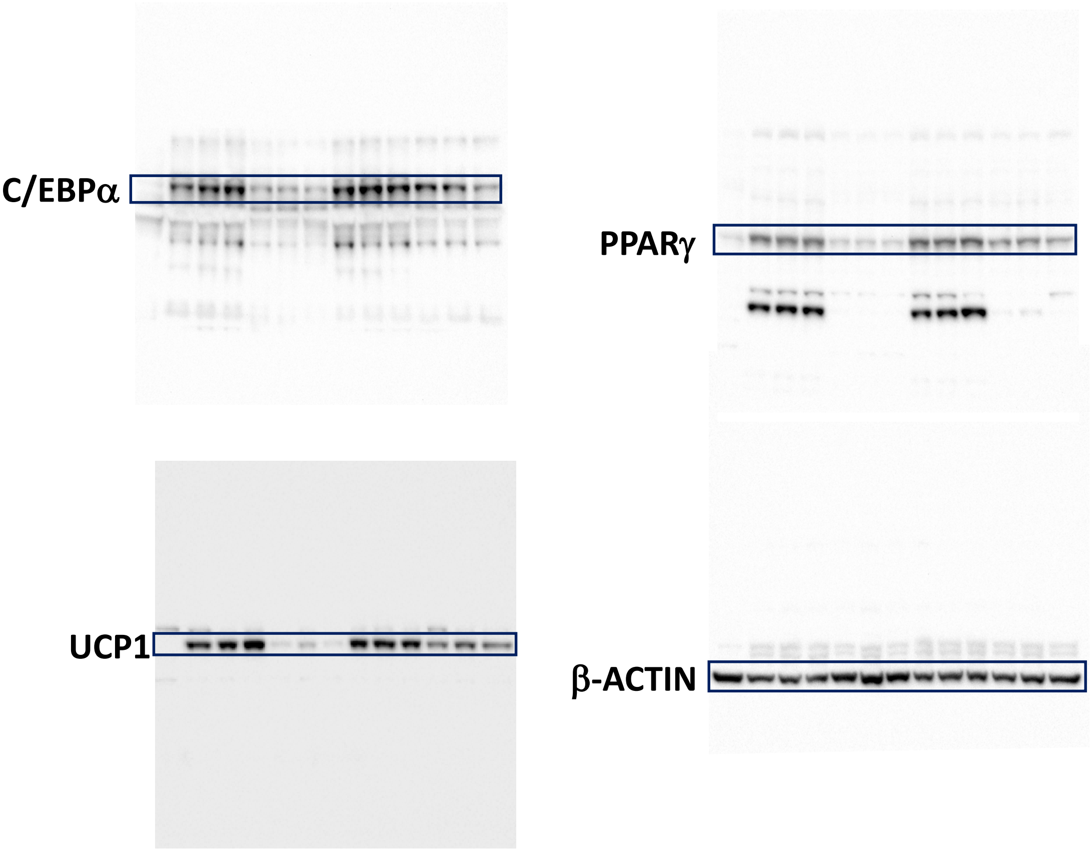

Fig. 8b, Uncropped images, middle 2 panels. The images shown in the middle 2 panels of (b), b-CATENIN and b-ACTIN, were obtained from top and bottom portions of the same blot, respectively.

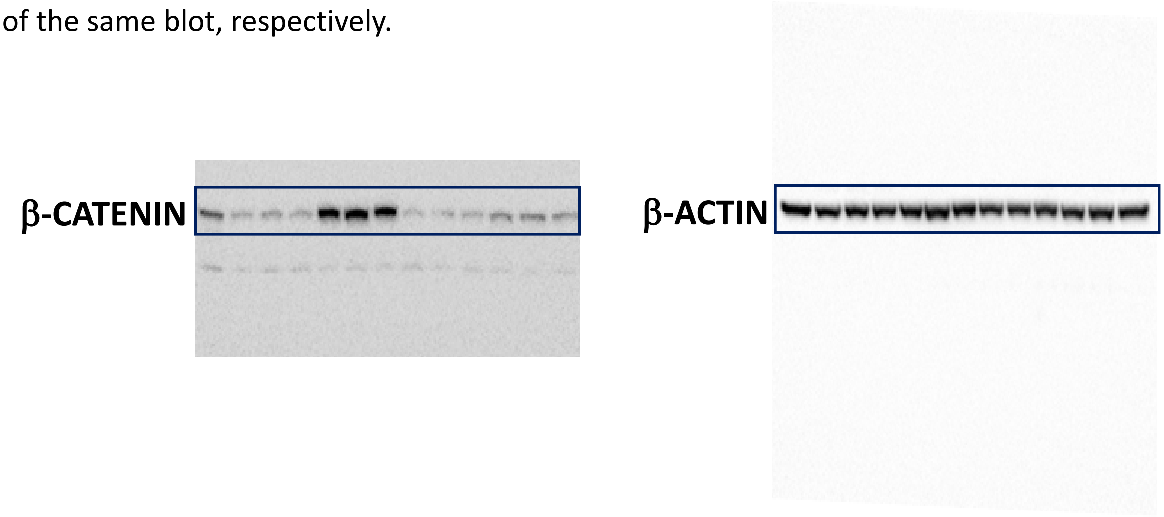

Fig. 8b. Uncropped images, bottom 3 panels. The images shown were obtained from the same blot. HSL was obtained from the top portion of the blot. ATGL and β-ACTIN were obtained from the bottom portion of the blot after stripping and re-probing.

ATGL

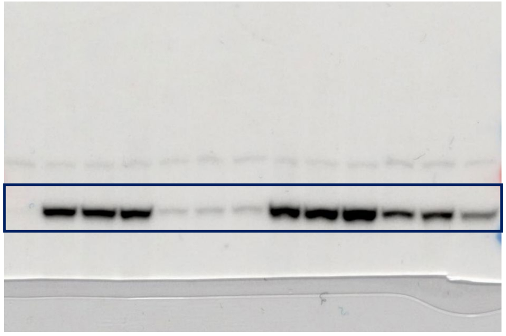

HSL

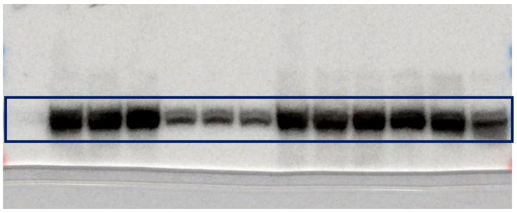

β-ACTIN

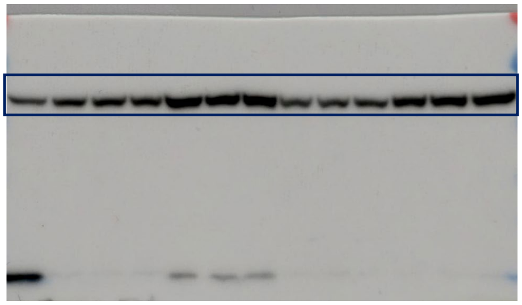

Fig. 8d. Uncropped images. The blot was cut and incubated with p-SMAD2 antibody. The same blot was then stripped and re-probed with SMAD-2 antibody.

p-SMAD2

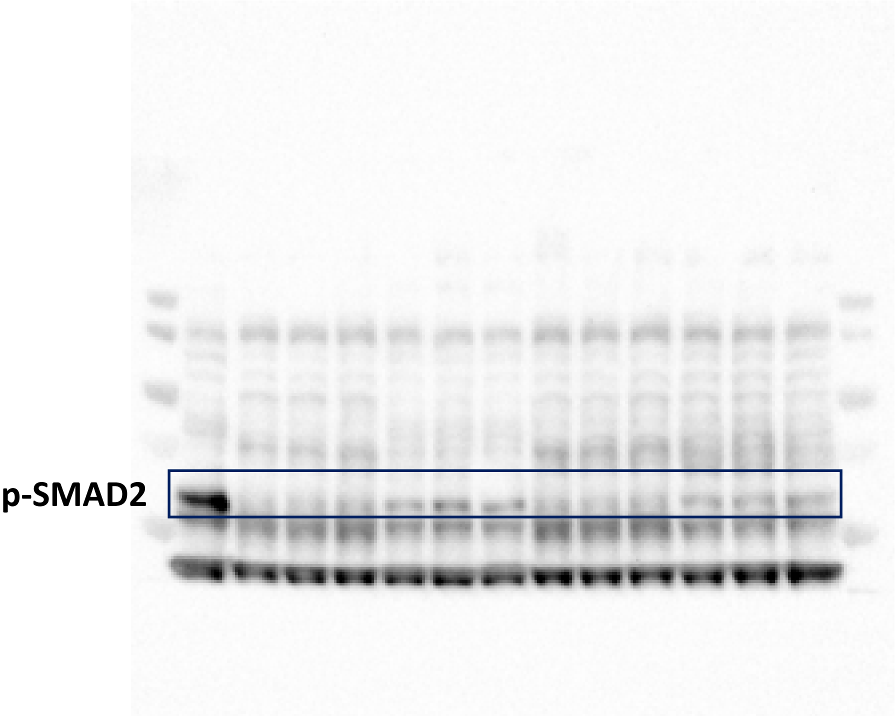

SMAD2

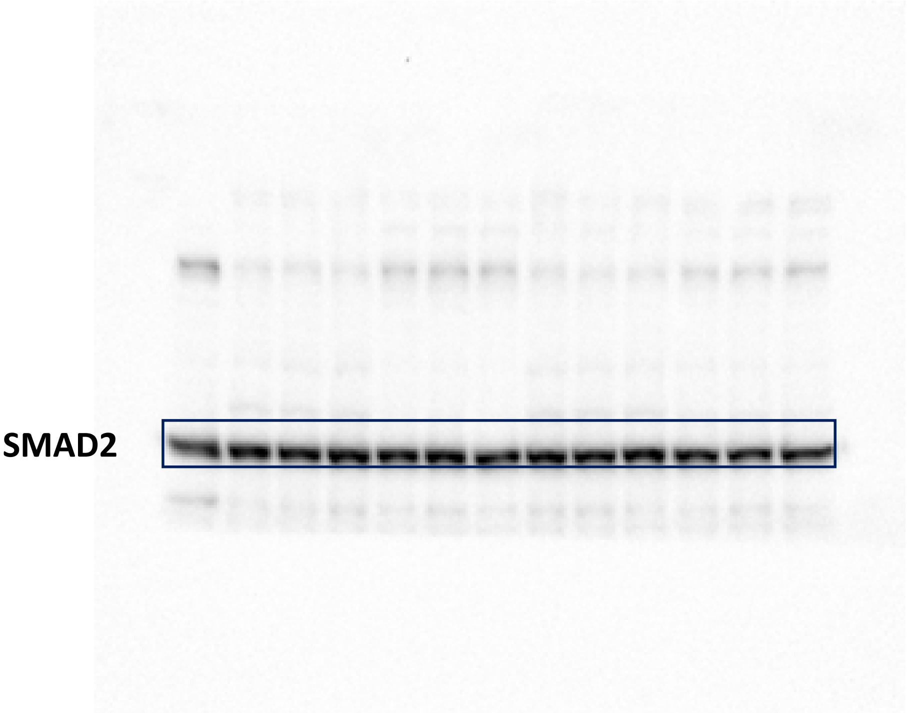

Supplement: Supplementary file 1 — Supplementary Information. [file 41598_2021_95720_MOESM1_ESM.pdf]
